# Museomics of tree squirrels: a dense taxon sampling of mitogenomes reveals hidden diversity, phenotypic convergence, and the need of a taxonomic overhaul

**DOI:** 10.1186/s12862-020-01639-y

**Published:** 2020-06-26

**Authors:** Edson Fiedler de Abreu-Jr, Silvia E. Pavan, Mirian T. N. Tsuchiya, Don E. Wilson, Alexandre R. Percequillo, Jesús E. Maldonado

**Affiliations:** 1 Laboratório de Mamíferos, Departamento de Ciências Biológicas, Escola Superior de Agricultura Luiz de Queiroz, Universidade de São Paulo, Piracicaba, SP 13418900 Brazil; 2 Center for Conservation Genomics, Smithsonian Conservation Biology Institute, National Zoological Park, Washington, DC, 20013 USA; 3 Coordenação de Zoologia, Museu Paraense Emílio Goeldi, Belém, PA 66077530 Brazil; 4 Data Science Lab, Office of the Chief Information Officer, Smithsonian Institution, Washington, DC 20560 USA; 5 Division of Mammals, National Museum of Natural History, Smithsonian Institution, Washington, DC 20560 USA; 6 Department of Biology and Department of Environmental Science and Policy, George Mason University, Fairfax, VA 22030 USA

**Keywords:** Historical DNA, Morphology, Neotropical region, Phylogeny, Sciuridae, Systematics

## Abstract

**Background:**

Tree squirrels (Sciuridae, Sciurini), in particular the highly diverse Neotropical lineages, are amongst the most rapidly diversifying branches of the mammal tree of life but also some of the least known. Negligence of this group by systematists is likely a product of the difficulties in assessing morphological informative traits and of the scarcity or unavailability of fresh tissue samples for DNA sequencing. The highly discrepant taxonomic arrangements are a consequence of the lack of phylogenies and the exclusive phenotypic-based classifications, which can be misleading in a group with conservative morphology. Here we used high-throughput sequencing and an unprecedented sampling of museum specimens to provide the first comprehensive phylogeny of tree squirrels, with a special emphasis on Neotropical taxa.

**Results:**

We obtained complete or partial mitochondrial genomes from 232 historical and modern samples, representing 40 of the 43 currently recognized species of Sciurini. Our phylogenetic analyses—performed with datasets differing on levels of missing data and taxa under distinct analytical methods—strongly support the monophyly of Sciurini and consistently recovered 12 major clades within the tribe. We found evidence that the diversity of Neotropical tree squirrels is underestimated, with at least six lineages that represent taxa to be named or revalidated. Ancestral state reconstructions of number of upper premolars and number of mammae indicated that alternative conditions of both characters must have evolved multiple times throughout the evolutionary history of tree squirrels.

**Conclusions:**

Complete mitogenomes were obtained from museum specimens as old as 120 years, reinforcing the potential of historical samples for phylogenetic inferences of elusive lineages of the tree of life. None of the taxonomic arrangements ever proposed for tree squirrels fully corresponded to our phylogenetic reconstruction, with only a few of the currently recognized genera recovered as monophyletic. By investigating the evolution of two morphological traits widely employed in the taxonomy of the group, we revealed that their homoplastic nature can help explain the incongruence between phylogenetic results and the classification schemes presented so far. Based on our phylogenetic results we suggest a tentative supraspecific taxonomic arrangement for Sciurini, employing 13 generic names used in previous taxonomic classifications.

## Background

Squirrels (Sciuridae) comprise the third most diverse family of rodents, with about 60 genera and 300 species organized in five subfamilies [1–3]. In the Neotropics, squirrels are inhabitants of all forest biomes [1, 2], and crucial to ecosystem dynamics as they play a vital role in seed predation and dispersal [4, 5]. However, in contrast to other widespread Neotropical rodent groups, squirrels have been largely neglected in phylogenetic studies. South American (SA) tree squirrels (tribe Sciurini) have been suggested as one of the most rapidly diversifying branches of mammals [6]. Still, the most representative phylogenetic hypothesis [7] included only nine samples representing less than one third of the SA species (sensu [2]) and it was based on a supermatrix of five genes with over 60% of missing data. As a result, basic knowledge on phylogenetic relationships is lacking for Neotropical lineages and their evolutionary history remains unraveled.

The lack of comprehensive phylogenies is likely a consequence of the difficulties in assessing phylogenetically informative morphological traits in a diverse group, with small sample sizes available in museums that are widely distributed throughout three continents [1]. This is also likely due to their conservative cranial morphology, as stated by Moore [21: 201]: “An enumeration and comparison of the taxonomic skull characters of the genera of Sciurinae reveal indications of great conservatism in genera occupying the typical tree squirrel niche, […], whereas genera occupying other sciurine niches appear to have had greater freedom to acquire skull character specializations”. Another very plausible reason for the long-term neglect of this diverse group by molecular systematists is the relative rarity of ethanol-preserved/frozen tissues available in scientific collections. In contrast to Nearctic squirrels, Neotropical forms are elusive, trap-shy, and generally restricted to well-preserved forests that can be difficult to access [8]. Conventional traps have proven inefficient to capture tree squirrels, while shotguns have been shown to be much more efficient [9]; although, due to logistical difficulties and permit regulations and restrictions for carrying firearms in many Latin American countries, their use has been virtually abandoned on SA small mammal surveys in the current century.

Historical tissue samples (e.g. dried tissue snipped from skins or scraped from skeletal material), on the other hand, can be more readily obtained from museum specimens—which were collected in early expeditions when the use of shotguns for scientific specimen collection was feasible and the commonest sampling method. However, not long ago, DNA derived from historical museum specimens was considered very difficult to obtain and inefficient to use in large scale phylogenetic analyses, due to the small quantities of degraded DNA that these samples yield, and genetic data was restricted to small fragments of a few mtDNA genes [10]. More recently, the techniques for obtaining whole mitogenomes from historical museum specimens (also called “Museomics”; see [11]) have undergone significant advances [12–14] and historical samples have been demonstrated to be a reliable and effective source of genetic data, especially if applied to next-generation sequencing methods (e.g. shotgun sequencing, targeted sequencing via hybridization-based captures or via restriction enzyme-based enrichment), which are very efficient in massively sequencing fragmented DNA [15–17].

Lack of knowledge of phylogenetic relationships resulted in highly discrepant taxonomic arrangements proposed for the group. In 1915, Joel A. Allen published the most comprehensive taxonomic revision of the SA squirrels. This work was the culmination of a couple of decades of impressive research describing and organizing the diversity of New World squirrels. Allen [18] recognized the tribe Sciurini as including eight genera and 32 species in SA, and he also recognized another 35 species in eight genera from Central America (CA) and North America (NA). Although no other comprehensive revisionary study has been published for this tribe since then, several subsequent authors have adopted different taxonomic arrangements that recognized two to four genera, and 12 to 14 species of Sciurini in SA (e.g. [1, 19–22]). More recently, Vivo and Carmignotto [2] published a new taxonomic proposal, where they recognize six genera and 18 species for SA Sciurini—a lower diversity when compared to that proposed by Allen [18], but greater when compared to subsequent authors, especially at the generic level. The huge discrepancy between the arrangements, all based exclusively on morphological data, attests to the extensive variation—and potential homoplastic nature—of the characters traditionally employed in taxonomy, and evidences the need for systematic reassessment using independent sources of information, such as genetic data.

Here we report the results of our analyses of mitochondrial genome data obtained from a combination of ethanol-preserved tissue samples (also referred to as “modern” samples) and tissue samples obtained from dry museum specimens (hereafter “historical” samples) representing most of the nominal taxa recognized as valid species of tree squirrels (Sciurini). We (1) provide the first phylogenetic hypothesis of Neotropical tree squirrels based on dense taxonomic sampling and on state-of-the-art methods of data generation and phylogenetic reconstruction; (2) contrast our results with generic arrangements proposed for the tribe and discuss the taxonomic implications; and (3) investigate the evolution of number of upper premolars and number of pairs of mammae—two morphological characters traditionally employed for taxonomic classification of tree squirrels. We take advantage of our large sampling across a diverse lineage of mammals to investigate how distinct aspects of historical samples (e.g. date of collection, museum of provenance, type of sample) might influence mitogenome recovery, and to provide useful information for future genetic studies sampling from dry museum specimens.

## Results

### Summary of mitochondrial genome sequencing success, assembly and synteny

From the 271 samples that we attempted to sequence, complete mitochondrial genomes were recovered for 92 samples, partial mitogenomes with variable percentages of missing data were recovered for 172 samples, and no mtDNA sequences were recovered for seven samples (Table 1). Modern samples yield, unsurprisingly, genomes more complete than historical samples, with full mitochondrial genomes recovered for almost half of modern samples (78 out of 177), but for only about 1/6 of historical samples (14 out of 94). Even though ethanol-preserved tissues yield more complete mitogenomes, we were able to obtain partial mitogenomes from most historical samples—for which ethanol-preserved tissues were not available—producing an increment of 18 nominal taxa to our phylogenetic datasets.

**Table 1:** Details on mitogenome completeness obtained in this study, including success for modern and historical samples

Percentage of mitogenome completeness	Number of samples	Number of modern samples and percentage of success	Number of historical samples and percentage of success
100%	92	78 (44.1%)	14 (14.9%)
100–80%	70	51 (28.8%)	19 (20.2%)
80–60%	24	11 (6.2%)	13 (13.8%)
60–40%	24	14 (7.9%)	10 (10.6%)
40–20%	22	10 (5.6%)	12 (12.8%)
< 20%	32	11 (6.2%)	21 (22.3%)
No coverage	7	2 (1.1%)	5 (5.3%)
**Total**	**271**	**177**	**94**

**Fig. 1:**
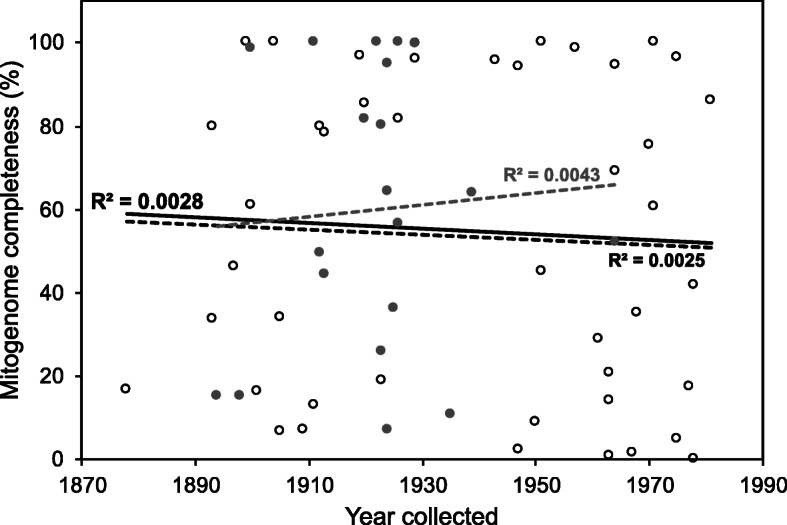
Relationship between specimen age and mitochondrial genome completeness recovered from historical samples of Sciurini. Open circles represent osteocrusts from the National Museum of Natural History (USNM; *N* = 44), while gray circles represent osteocrusts from the American Museum of Natural History (AMNH; *N* = 20). Dashed black line represents linear regression based on samples from USNM and dashed gray line from ANMH, while the solid line represents the linear regression based on the samples from both collections. None of the linear regressions performed were significant (*P* > 0.05)

When contrasting mitogenome recovery success (completeness) of historical samples with tissue type and museum location, we note that, on average, remains of muscular tissue adherent to skulls (“osteocrusts”) yielded more complete mitogenomes than skin clips, and samples from NA collections yielded more complete mitogenomes than samples from SA collections, although none of those differences were significant (X^2^ = 60.114, *P* = 0.6146 and X^2^ = 68.398, *P* = 0.3304, respectively; Additional file 1). We found no correlation between sample age (the year in which the specimen was collected) and completeness of mitogenomes recovered for historical samples (R^2^ = 0.0028, *P* = 0.6762; Fig. 1). We were able to obtain complete mitogenomes for specimens as old as 120 years, and partial mitogenomes with over 20% of completeness for specimens as old as 126 years.

**Fig. 2:**
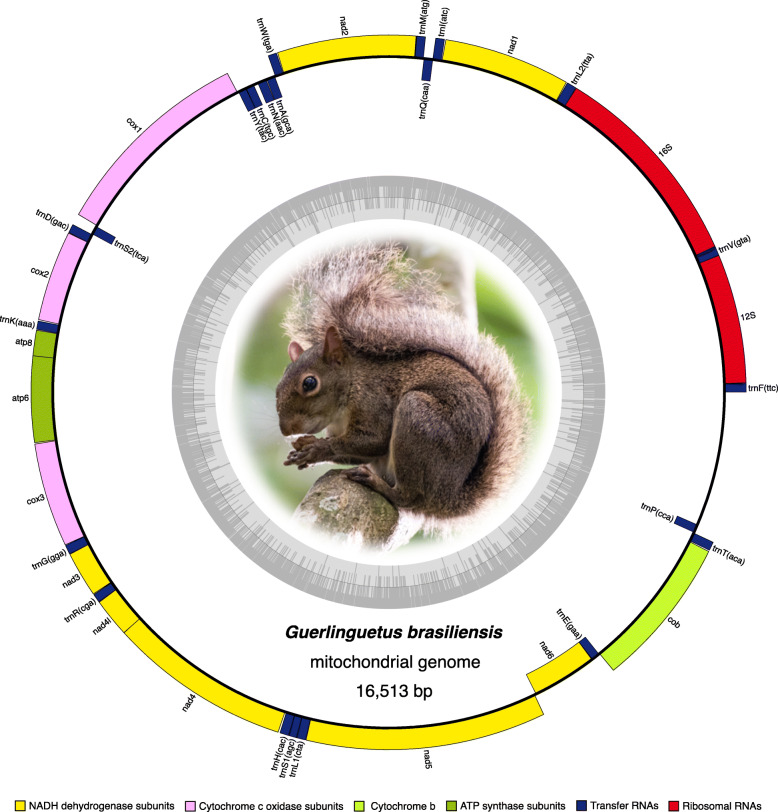
Circular mitochondrial genome map of *Guerlinguetus brasiliensis* depicting the gene organization in tree squirrels. The inner circle shows the GC content along the mitogenome. Photograph of *G. brasiliensis* by Pedro Peloso

The complete assembled mitogenomes were circular molecules with length ranging from 16,501 to 16,535 pb. For all species analyzed, including ingroup (tribe Sciurini) and outgroup (tribe Pteromyini) taxa, mitogenomes presented identical synteny, comprising 13 protein-coding genes (PCGs), two ribosomal RNA genes (rRNA), and 22 transfer RNA genes (tRNA). The GC-content ranged from 36.6 to 38.9%. The annotated circular genome of *Guerlinguetus brasiliensis* is depicted in Fig. 2 to exemplify the gene organization in the group. This is the first complete mitochondrial genome published for a Neotropical squirrel.

### Phylogenetic inferences and the effect of missing data

**Fig. 3:**
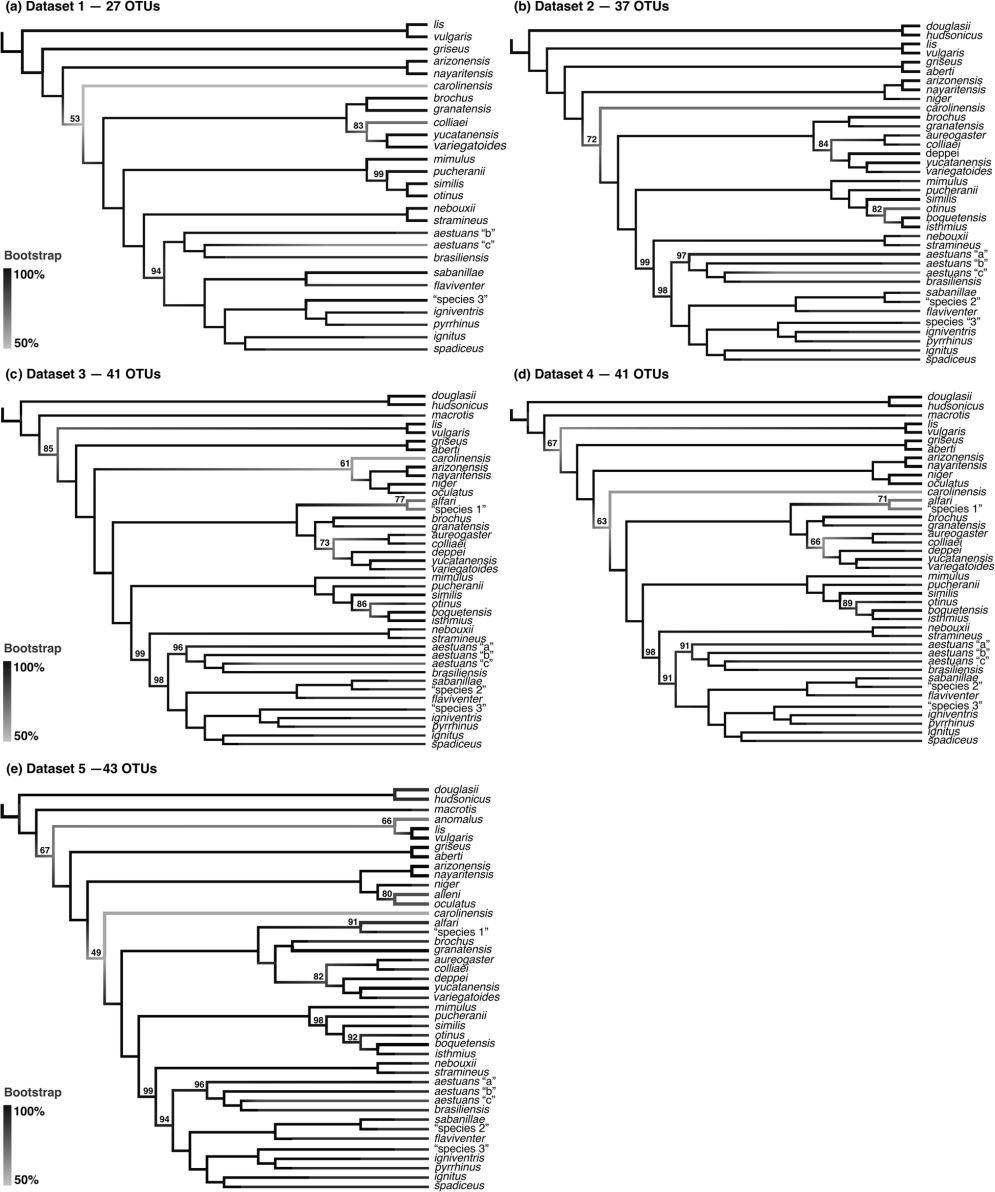
Simplified ML trees of Sciurini based on analyses of five distinct datasets. **a** Dataset 1—92 specimens with no missing data, **b** Dataset 2—162 specimens with < 20% of missing data per sample, **c** Dataset 3—186 specimens with < 40% of missing data per sample, **d** Dataset 4—210 specimens with < 60% of missing data per sample, and **e** Dataset 5—232 specimens with < 80% of missing data per sample. Additional details of each dataset are provided in Table 2. Numbers above branches indicate support values for all nodes that presented bootstrap frequencies below 100%

The resulting matrices of Datasets 1–5 included 92 to 232 specimens representing 27 to 43 Operational Taxonomic Units (OTUs). This represents the most conservative hypothesis at the species level that reconciles the initial morphological identifications and our phylogenetic results—i.e. monophyletic groups (see OTUs designation in the section “Species monophyly and species recognition”). The overall missing data ranged from 0.1–20.2%. Characteristics of each of the five matrices with different levels of missing taxa and data are summarized in Table 2. Comparisons of phylogenetic trees inferred from those datasets does not indicate that the inclusion of specimens with partial mitogenomes (missing data) produced any strong topological incongruences (Fig. 3). Except for the phylogenetic position of *Neosciurus carolinensis* (see details below), the topologies recovered with Datasets 1–5 are similar. Most of the nodes were strongly supported in all of our dataset analyses, and the inclusion of specimens with up to 80% missing data had no overall impact on the nodal support of the inferred ML phylogenies, as there were no significant differences on the average Bootstrap values recovered (Wilcoxon signed-rank test *P* > 0.05).

**Table 2:** Summary features of the mitogenome datasets analyzed in this study

	Dataset 1	Dataset 2	Dataset 3	Dataset 4	Dataset 5
Mitogenome completeness per specimen	100%	≥80%	≥60%	≥40%	≥20%
Minimum mitogenome length per specimen	16,376 bp	12,099 pb	7144 bp	3884 pb	2118 pb
Number of specimens included	92	162	186	210	232
Alignment overall missing data	0.1%	3.7%	8.3%	14.2%	20.2%
Number of OTUs included	27	37	41	41	43
Average nodal support (bootstrap) of the inferred ML trees	97.4	98.2	97.0	96.1	95.8

**Fig. 4:**
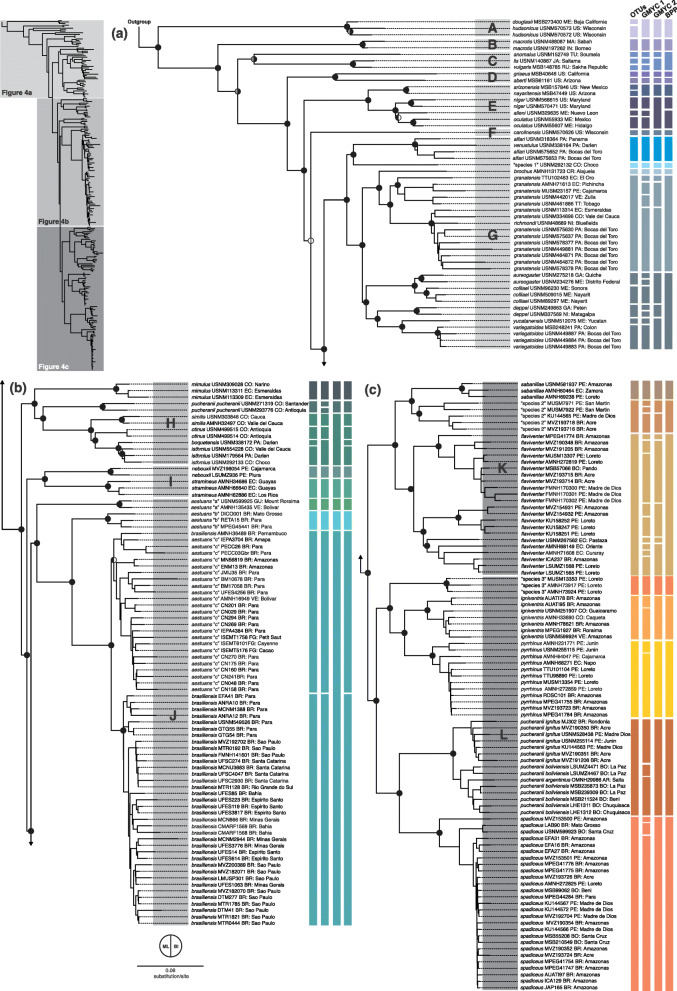
(parts **a, b,** and **c**). Mitochondrial phylogenomic inference of Sciurini recovered by ML analysis of 232 specimens (Dataset 5). Nodal support from the ML bootstrap pseudo-replicates are indicated at each node along with Bayesian posterior probabilities (BI). White wedges indicate bootstrap values ≤ 50%, grey indicates bootstrap frequencies between 50 and 75%, and black indicates bootstrap frequencies ≥ 75%. For BI, white indicates PP < 0.95, whereas black indicates PP ≥ 0.95. Scale at the bottom represents substitutions per site. Letters A–L identify species groups discussed in the text. Except by putative unnamed species (“species 1–3”), terminals are named with specific epithets following [1, 2], and [24] (see methods for details), accompanied by museum voucher numbers and geographic information (country code and state/department). The first column represents Operacional Taxonomic Units (OTUs), recognized with base on original identifications and monophyly. The second to fourth columns correspond to the status of the delimitation of OTUs by different species delimitation methods: generalized mixed Yule-coalescent models using ultrametric trees generated with strict molecular clock (GMYC 1) and relaxed log-normal clock (GMYC 2); and Bayesian Phylogenetics and Phylogeography (BPP) analyses

Considering the aforementioned results, and that including as many specimens as possible would be the best option to unveil all mitochondrial lineages, the tree recovered by the Maximum Likelihood (ML) analysis of Dataset 5 (the most taxonomically comprehensive dataset, with 232 specimens) was chosen to represent the mitochondrial phylogenetic hypothesis of Sciurini. This hypothesis is shown in detail in Fig. 4 with accompanying nodal support from bootstrap replicates. Bayesian inference of Dataset 5 recovered a topology similar to the one recovered by ML analysis of the same matrix. Best-fitting models of sequence evolution used on the BI are summarized in Additional file 2. Nodal support recovered as posterior probabilities by the Bayesian Inference (BI) of Dataset 5 is also shown in Fig. 4.

Since our various analyses including different numbers of taxa recovered similar topologies, our results support essentially the same conclusions discussed below. Most of the differences recovered are related to the sampling difference, when species represented in some datasets were not represented in others. Any inconsistencies recovered by the different analyses are mentioned below, when appropriate.

### A mitogenomic hypothesis: Sciurini and its species groups

The genus-level classification of Sciurini is greatly discordant among all previous taxonomic arrangements proposed for the group, and none of those classifications fully matched the phylogenies obtained by our optimality criteria. Therefore, to avoid more confusion on the use of genus-level names, we deliberately omit the generic epithet of all taxa when describing our results below, and refer the species as members of species groups (identified by capital letters), as recovered by our phylogenetic analyses. A tentative genus-level classification of Sciurini is presented in the discussion (see “Mitochondrial phylogeny and taxonomic arrangements proposed for Sciurini”), supported by our results and by other relevant taxonomic information.

Our results recovered the tribe Sciurini as a monophyletic group with full nodal support. Within Sciurini we recognize 12 major groups, A–L (Fig. 4), that have been consistently recovered by all of our analyses where they were represented (Fig. 3). Except for Group F, which is composed of a single specimen, the groups recognized here represent clades, all of which were recovered with high statistical support on our inferred phylogenies (ML bootstrap ≥ 75% and BI posterior probability ≥ 0.95).

Group A is represented in our analyses by the North American nominal-taxa *hudsonicus* Erxleben, 1777 and *douglasii* Bachman, 1839. Group B is monotypic, consisting of *macrotis* Gray, 1856 from Borneo. Group C includes Eurasian radiations: *anomalus* Gmelin, 1778, *lis* Temminck, 1844, and *vulgaris* Linnaeus, 1758. The three subsequent groups contain mostly North American lineages, some of which also reach Central America: Group D includes *aberti* Woodhouse, 1852 and *griseus* Ord, 1818; Group E includes *arizonensis* Coues, 1867, *nayaritensis* J. A. Allen, 1890, *niger* Linnaeus, 1758, *alleni* Nelson, 1898, and *oculatus* Peters, 1863; and Group F is composed of a single representative of *carolinensis* Gmelin, 1788. While this taxon is broadly distributed in the eastern and midwestern USA, our sample comes from the midwestern USA (Fig. 4a).

**Fig. 5:**
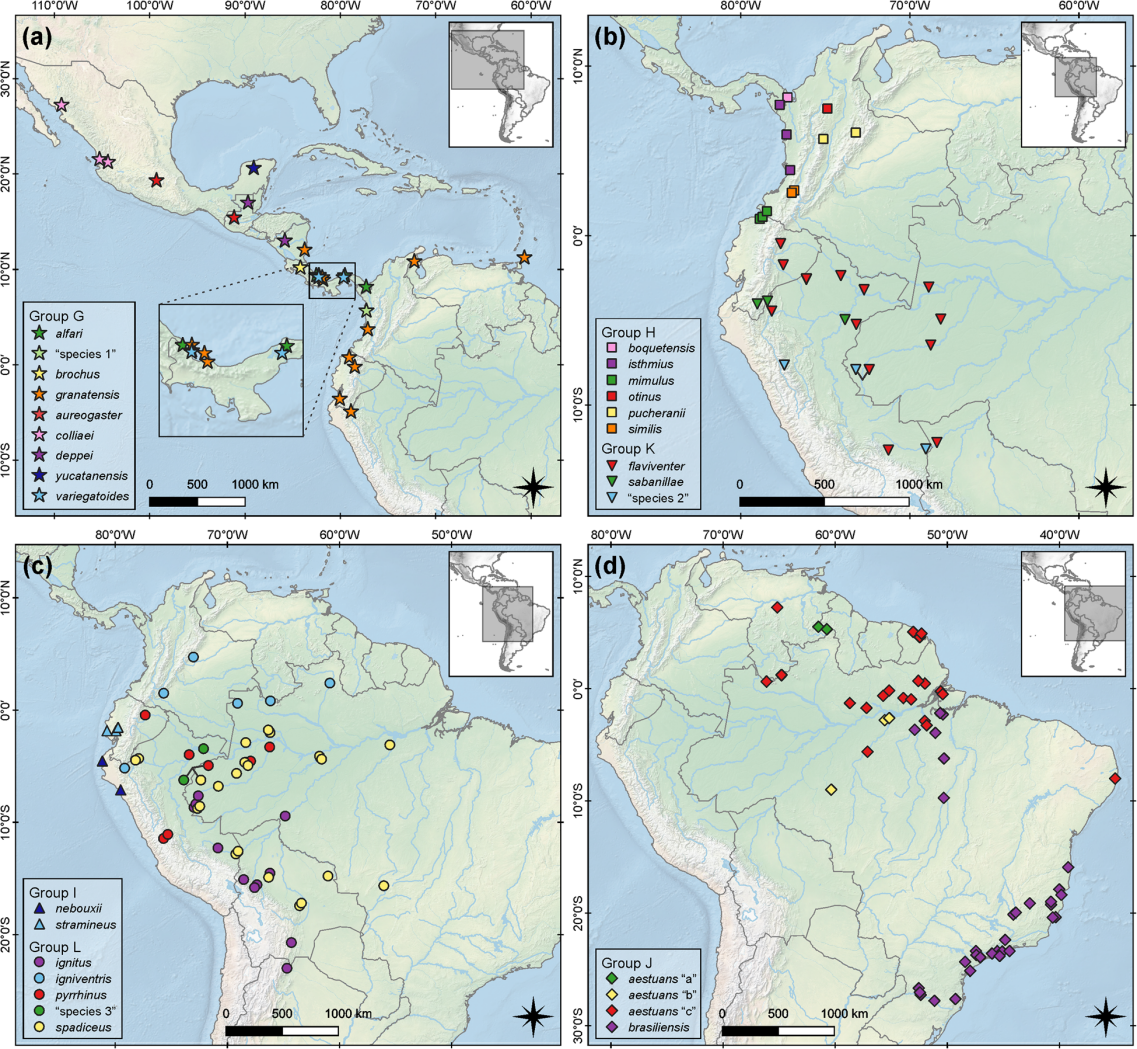
Collecting localities of samples composing Neotropical Sciurini lineages. Maps were generated using QGIS 3.4.4 (http://qgis.org)

Groups G–L contain all Neotropical lineages. Group G is composed of southern North American, Central American, and northern trans-Andean South American forms currently assigned to at least nine species or species complexes (Figs. 4a and 5a). This geographically and taxonomically inclusive clade contains the nominal taxa *alfari* J. A. Allen, 1895 from Panama, *venustulus* Goldman, 1912 from Panama*, brochus* Bangs, 1902 from Costa Rica, *granatensis* Humboldt, 1811 from Peru, Ecuador, Colombia, Panama, Venezuela, Trinidad and Tobago, and Nicaragua, *richmondi* Nelson, 1898 from Nicaragua, *aureogaster* F. Cuvier, 1829 from Guatemala and Mexico, *colliaei* Richardson, 1839 from Mexico, *deppei* Peters, 1863 from Guatemala and Nicaragua, *yucatanensis* J. A. Allen, 1877 from Mexico, and *variegatoides* Ogilby, 1839 from Panama. Besides these taxa, Group G includes a highly divergent lineage composed of a single specimen from Chocó, Colombia, which could not be assigned to any valid species (“species 1” in Fig. 4a).

Groups H–L are exclusively composed of specimens from South America and southern Panama (Figs. 4b–c and 5b–d). Group H comprises specimens from mountain areas in northwestern South America allocated to six species according to Vivo and Carmignotto [2]: *mimulus* Thomas, 1898 with specimens from Colombia and Ecuador; *similis* Nelson, 1899, *otinus* Thomas, 1901 and typical *pucheranii* Fitzinger, 1867 from Colombia; *boquetensis* Nelson, 1903 from Panama; and *isthmius* Nelson, 1899 from Colombia and Panama. Group I includes two species: *nebouxii* I. Geoffroy St. Hilaire, 1855 from the coast of Ecuador, and *stramineus* Eydoux and Souleyet, 1841 from the coast of Peru.

Group J is composed of specimens distributed throughout the Atlantic Forest along the eastern coast of Brazil, and in Amazonia including Brazil, French Guiana, Guyana, and Venezuela. This clade includes two nominal taxa: *aestuans* Linnaeus, 1766 and *brasiliensis* Gmelin, 1788. Group K includes two named species: *flaviventer* Gray, 1867 from Amazonian lowlands of Brazil, Bolivia, Ecuador, and Peru; and *sabanillae* Anthony, 1922 including samples from the Amazonian lowlands of Peru to the eastern Andean mid-elevations of Ecuador. Additionally, Group K includes what seems to be another unnamed lineage, composed of specimens from the Brazilian and Peruvian Amazon, and from mid-elevation of the eastern Andes in northern Peru (“species 2” in Fig. 4c).

Group L includes specimens associated with *igniventris* Wagner, 1842, *pyrrhinus* Thomas, 1898, and *spadiceus* Olfers, 1818 from lowland Amazonia in Brazil, Bolivia, Peru, Ecuador, and Colombia, and a putative unnamed lineage composed of three specimens from Loreto, Peru (“species 3” in Fig. 4c). In addition to those, Group L includes specimens assigned to *pucheranii* Fitzinger, 1867 by Vivo and Carmignotto [2] as subspecies: *pucheranii ignitus*—specimens from Brazil and Peru—, *pucheranii argentinius*—samples from Argentina— and *pucheranii boliviensis*—samples from Bolivia.

Our analyses recovered similar relationships amongst the 12 major groups within Sciurini, apart from Group F. This lineage, represented in our analyses by a single specimen of *carolinensis*, was recovered as sister to Group E by the ML analysis of Dataset 3 (Fig. 3c) and the BI of Dataset 5 (not shown), or as sister to clades G–L (all Central and South American squirrels) in all remaining analyses (Fig. 3a, b, d, e). However, the phylogenetic position of Group F was weakly supported (bootstrap < 75%, posterior probability < 0.95) in all our inferences. Amongst the intergroup relationships unanimously recovered by our analyses with high statistical support, we highlight the monophyly of the Neotropical forms (Groups G–L) and of the group that exclusively includes squirrels from South America and southern Panama (Groups H–L).

Except for comparisons between datasets with unrepresented taxa, interspecific and intraspecific relationships within the multispecies groups of Sciurini were similar in all ML analysis performed (see Fig. 3). Bayesian analysis of Dataset 5 also recovered similar results, apart from relationships within Group E, where the node for *alleni*, *oculatus* and *niger* was unresolved in a polytomy sister to *nayaritensis* + *arizonensis*.

### Species monophyly and species recognition

Our phylogenetic results corroborate most currently recognized species of Sciurini as highly supported clades (when represented by more than one specimen). However, some valid species appear nested within others, while other nominal taxa seem to include several distinct genetic lineages that do not comprise a monophyletic group. For example, a specimen originally identified as *richmondi* (USNM 48689) is nested within the clade associated with *granatensis* (Fig. 4a), an individual assigned to *venustulus* (USNM 338164) is nested within the clade associated with *alfari* (Fig. 4a), specimens identified as *pucheranii* (USNM 271319 and USNM 293776 in Group H, and MJ302–LHE1312 in Group L) are recovered in two phylogenetically distant lineages (Fig. 4a and c), and samples originally identified as *aestuans* (USNM 599925–CN158 in Group J) and *brasiliensis* (AMNH 36489 and EFA41–MTR0444 in Group J) do not compose reciprocally monophyletic groups (Fig. 4b).

Based on those results, we reassigned the 232 specimens used in our phylogenetic inferences to OTUs, i.e. monophyletic groups (shown along Fig. 4, first column). The most relevant differences between our OTU designations and the original identifications based on Thorington et al. [1] and Vivo and Carmignotto [2] are the recognition of: a single OTU (*granatensis*) for *granatensis* and *richmondi*; a single OTU (*alfari*) for *alfari* and *venustulus*, two distinct OTUs (*pucheranii* and *ignitus*) for *pucheranii*; and three OTUs (*aestuans* “a”, *aestuans* “b”, *aestuans* “c” [including the specimen from Pernambuco, AMNH 36849]) for samples originally assigned to *aestuans*. Additionally, our phylogenetic analyses recovered some apparently unnamed lineages that we also consider as distinct OTUs, referred to as “species 1” (from Group G), “species 2” (from Group K), and “species 3” (from Group L; Fig. 4). In total, we recognized 43 OTUs, some of which are composed of deeply divergent mitochondrial lineages that seem to merit further investigation (e.g. *granatensis*, *flaviventer* and “species 2”).

As the specific status of some nominal taxa seems questionable, and some apparently unnamed species were suggested by our phylogenetic inferences, we used species delimitation analyses to provide a quantitative evaluation of the species limits within Sciurini. GMYC analyses using as input ultrametric trees generated with strict (GMYC 1) and relaxed (GMYC 2) molecular clocks resulted in highly distinct species scenarios, suggesting 66 and 39 species within Sciurini, respectively. GMYC 1 analysis failed to recognize six OTUs as distinct species, four from North America—(*douglasii*, *hudsonicus*), (*arizonensis*, *nayaritensis*)—and two from South America—(*boquetensis*, *isthmius*). This analysis also suggested an additional 26 putative species (Fig. 4), aside from the original 43 OTUs.

GMYC 2 analysis provided a more conservative scenario and did not recognize 13 Eurasian, North American, and Central American OTUs—(*douglasii*, *hudsonicus*), (*lis*, *vulgaris*), (*arizonensis*, *nayaritensis*), and (*niger* (*alleni*, *oculatus*)), (*aureogaster*, *colliaei*) and (*yucatanensis*, *variegatoides*)—and six South American OTUs—(*similis* (*otinus* (*boquetensis*, *isthmius*))) and (*aestuans* “c”, *brasiliensis*) as distinct species. On the other hand, this analysis suggested additional seven putative species in three species complexes associated with the Neotropical OTUs *granatensis*, “species 2”, and *flaviventer* (Fig. 4).

BBP analysis recovered full support (PP = 1) for the recognition of 29 species and did not recover significant support (PP > 0.95) for the recognition of 14 species. All of the non-supported species are from Eurasia, North and Central Americas and are included in five lineages: (*douglasii*, *hudsonicus*), (*anomalus*, *lis*, *vulgaris*), (*arizonensis*, *nayaritensis*), (*alleni*, *oculatus*) and (*aureogaster*, *colliaei*, *deppei*, *yucatanensis*, *variegatoides*).

Despite the differences observed in the results of species delimitation analyses, the most noteworthy result was the consistent support of all South American OTUs as distinct species by at least two analyses. The only exception is *boquetensis* and *isthmius*, which were not supported as distinct species by the GMYC analyses using both strict and relaxed clock generated chronograms, but they were recovered as distinct species with full support by BPP analysis. On the other hand, some Eurasian, North American and Central American OTUs were consistently not supported as distinct species (see Fig. 4). For the taxa of non-South American lineages, we have a limited number of specimens in our analysis (one specimen per OTU in most cases), and this might be blurring the species delimitation analyses.

**Fig. 6:**
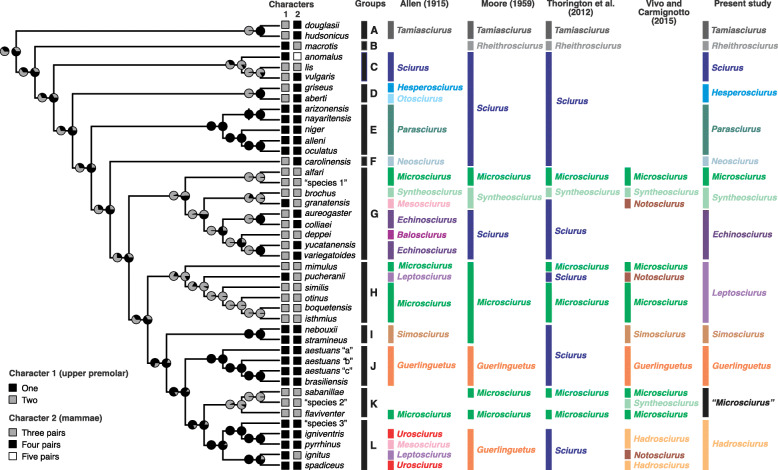
Ancestral state reconstructions of morphological characters and comparison between phylogeny and classification schemes of Sciurini. The first column identifies species groups (**a**–**l**) discussed in the text, the second to fifth columns show previous classifications of Sciurini at genus level, and the last column depicts the tentative generic classification suggested by this study. Morphological characters are number of premolars (character 1) and number of pairs of mammae (character 2). See text for character definitions and scoring details. Pie diagrams at internal nodes represent estimated probabilities of alternative states

Based on our phylogenetic inferences and species delimitation analyses, as well as available information in the literature (e.g. phenotypic and karyotypic data, analyses of geographic variation and previous phylogenetic and species delimitation analyses [1, 2, 24–28]), we recognized the initial set of 43 OTUs as putative species of Sciurini (Fig. 6), all of which are treated as distinct terminal taxa in the following analyses. From those 43 OTUs, 37 represent described species recognized as valid by the latest taxonomic hypothesis comprising those taxa ([1, 2, 24]; but *granatensis* includes *richmondi* and *alfari* includes *venustulus*), while six are putative additional species to be described or revalidated (“species 1–3”, *aestuans* “a–b”, and *ignitus*—currently considered as a subspecies of *pucherani* by [2], but meriting specific status as per our results). For detailed justification for taxonomic decisions and name usage, please see the discussion section “Comments on species recognition and novelties”.

### The evolution of premolars and mammae within Sciurini

The best-fitting model to explain the evolution of number of premolars within Sciurini was the Mk with equal rates (AICw = 0.686; Additional file 3), which suggests that transitions between states occurred at the same rate and with equal probabilities. For the number of pairs of mammae, the most supported model was Mk with symmetric rates (AICw = 0.763; Additional file 4), suggesting that changes between states had equal probabilities regardless of direction, but differed in rates. Ancestral state reconstructions suggest that the most recent common ancestor (MRCA) of Sciurini had, most likely, two upper premolars (*P* = 0.53) and four pairs of mammae (*P* = 0.56; Fig. 5). However, alternative states for both characters were recovered with nearly equal probabilities for this node, indicating that the MRCA of Sciurini might also have had one upper premolar (*P* = 0.47) and three pairs of mammae (*P* = 0.44). Despite the uncertainty regarding the deepest nodes of Sciurini, several of the major clades recognized within the tribe exhibited unambiguous optimizations, indicating that conditions of both characters must have evolved multiple times during the evolutionary history of tree squirrels.

Two upper premolars were likely (*P* > 0.70) for the MRCA of six major Groups (A, C, D, G, H, and K), while one upper premolar was likely (P > 0.70) for the MRCAs of other four Groups (E, I, J, and L). Considering the estimates within the major Groups of Sciurini, the loss of one upper premolar seem to have happened at least three independent times (*anomalus* in C, *granatensis* in G, and *pucheranii* in H). On the other hand, the MRCA of the clade formed by Groups I, J, K, and L likely had one premolar (*P* > 0.70), and the condition of Group K, two premolars, could be interpreted as a new gain or a return to an ancestral state. Regarding the number of mammae, three pairs were likely (P > 0.70) present on the MRCA of four Groups (C, G, H, and K), while four pairs were likely (P > 0.70) present on the MRCA of six Groups (A, D, E, I, J, and L). Considering the estimates within the major Groups of Sciurini, several changes in the number of pairs of mammae are evident, with at least three independent changes from three to four/five pairs (*anomalus* and *vulgaris* in C, and MRCA of *aureogaster*, *colliaei*, *deppei*, *yucatanensis*, and *variegatoides* in G) and two independent transitions from four to three pairs (*deppei* in G and *ignitus* in L).

## Discussion

### The importance of museum specimens for the study of Neotropical tree squirrels

All samples used on this study were gathered from specimens deposited in scientific collections. The inclusion of historical samples was crucial in the detection of several taxonomic issues reported here. Almost a third of our samples were obtained from dry museum specimens, collected between 1893 and 2010, and housed mostly in two North American museums (AMNH and USNM). We were successful in obtaining at least 20% of the mitogenome for about 70% of the historical samples, which allowed us to include 18 nominal taxa in the Sciurini phylogeny for which ethanol-preserved tissues were not available. Moreover, two of the main groups recognized within Sciurini (B and H) were exclusively represented by historical samples.

Our success in obtaining mtDNA data from historical samples is twofold: i) it could be partially attributed to the sequencing method employed. Next-generation sequencing techniques (e.g. shotgun sequencing, targeted sequencing via hybridization-based captures or via restriction enzyme-based enrichment [30]) are highly advantageous for obtaining large-scale genomic data from historical samples in comparison with traditional sequencing methods (e.g. Sanger sequencing), as they are very efficient in sequencing short fragments of DNA [14], which are expectedly abundant in old museum samples; ii) it could also be a consequence of our sampling strategy. We prioritized obtaining fragments of muscular tissue adhered to skulls, which had been shown by previous studies [12] and confirmed here, to yield higher concentrations and longer fragments of DNA than samples obtained from skins clips.

The use of historical samples in molecular studies has increased substantially in the last two decades (see review in [14, 31]) and is helping to reveal hidden diversity, to unveil puzzling phylogenetic relationships, and to place rare and elusive mammal species/lineages in a phylogenetic context [16, 32–34], including in squirrels [35]. For Neotropical mammal groups, the study of historical museum samples using high-throughput sequencing to address phylogenetic and taxonomic questions is growing, becoming more feasible, and holding lots of promise [36, 37].

When investigating how different aspects of historical samples of Sciurini might influence the success of mitogenome recovery, our most robust result was that sample age (the year in which the specimen was collected) does not affect the completeness of mitogenomes obtained (Fig. 1). Previous studies that compared age of source sample with other metrics of mitochondrial DNA recovery (e.g. copy number) also found no relationship between those two factors [12, 15, 38]. Together, those findings highlight the potential of old museum specimens—including holotypes and other taxonomically important material—for phylogenetic and evolutionary inferences. Future studies on Neotropical mammals could benefit from the large series of specimens collected in South America during the first decades of the twentieth century (e.g. by A. Garbe, see [39]; and by the Olalla family, see [40]). These valuable and irreplaceable specimens—many of those from localities that were long-ago transformed into human modified landscapes—will allow the reconstruction of comprehensive phylogenetic hypotheses for taxonomic groups for which samples of fresh, frozen, or ethanol-preserved tissue are absent, scarce, and/or difficult to obtain.

Despite the immense value of historical material, as aforementioned, we advocate that this type of sample should be used as a complement to traditional ethanol-preserved samples. As documented by our and previous studies [12], modern samples result in higher sequencing success, and they have the great advantage of not being destructive, in any sense, to the morphological vouchers. Therefore, whenever possible, they should be preferred as a primary source of genetic data. In this sense, contemporary field sampling using diverse collection techniques is crucial to increasing the representativeness and value of our repositories of biodiversity, as defended by Voss and Emmons [9]. The continuity of field expeditions, especially to remote and unsampled regions to collect new specimens, will certainly result in the discovery of many unknown species, which is imperative to uncover the still hidden biodiversity of the richest areas of the globe, including the Neotropics [41]. Ultimately, while historical specimens are a critical and essential resource, they should not obscure the potential value of obtaining additional specimens for science in the wild, which are paramount not only for taxonomic and phylogenetic refinement, but also for documenting ongoing ecological and evolutionary changes and promoting biodiversity conservation.

### Missing data versus missing taxa

The discussion regarding how much missing data (and their effects) should be allowed in phylogenetic inferences has gained considerable attention, especially after the dissemination of next-generation sequencing methods (e.g. [29, 42–44]). It was previously shown that missing data could obscure phylogenetic relationships and promote negative impacts on the phylogenies (e.g. [45]). Subsequent authors (e.g. [43]) have shown that including as many loci as possible in the phylogenetic analyses is benefical even with large amounts of missing data because this would increase the sampling of distinct regions across the genome. Streicher et al. [44] emphasized that the optimal approach in terms of amount of missing data incorporated without losing the accuracy of the inference depends on the dataset and the phylogenetic method employed. After exploring our data by comparing the performance of matrices with alternative sampling strategies, we observed that the addition of samples with a limited amount of missing data did not impact the estimated relationships nor cause significant change to the nodal support of the inferred phylogenies.

### Mitochondrial phylogeny and taxonomic arrangements proposed for Sciurini

The comparison of our results with proposed taxonomic arrangements for Sciurini ([1, 2, 18, 21] which is identical to [20, 22]) illustrates that none of the generic arrangements fully corresponds to the phylogenetic structure recovered (Fig. 5). Most currently recognized genera (by [1, 2]) are not recovered as monophyletic by our analyses of genetic data. Allen [18] suggested the greatest diversity of genera, and his hypothesis seems to be the one that best fits our results, especially regarding the Nearctic taxa.

The delimitation of the taxa at the genus-group level has not received as much attention as species delimitation [46, 47]. Recently published generic arrangements for South American rodents, which included the description of new genera [23, 48, 49], have provided a solid diagnosis for the new taxa by consistently testing phylogenetic hypotheses and employing reciprocal monophyly as a primary criteria. Their phylogenetic analyses were complemented with robust and consistent morphologic analyses and they then applied total evidence analysis or conducted a posteriori comparison to support taxon diagnosis.

Here we use the phylogenetic information provided by a taxonomically robust mitogenome dataset to suggest a tentative classification at the genus level for Sciurini (Fig. 6). In our arrangement, we recognize reciprocal monophyletic entities as taxa at the genus-group level, and most of those entities correspond to groups A to L as recovered by our analyses; the only exception is the recognition of three reciprocally monophyletic genera within Group G, based on previous classificatory arrangements [1, 2, 18, 21]. We attribute to them the appropriate available names following the criteria established by the International Commission on Zoological Nomenclature (ICZN). Therefore, we favor the principles of priority and stability in such tentative nomenclatural acts, employing whenever possible the generic names proposed by Allen in 1915 [18], the first reviewer and author of several names of the genus-group valid and available for this radiation of squirrels.

For the sake of consistency with current generic nomenclatural acts (see above), our limited morphologic dataset (number of pairs of mammae and of upper premolars) precludes us from providing formal diagnosis and description for a presumptive taxon, a goal that is beyond the scope of this contribution. Thus, for the lineage of the genus-group level with no available name, we apply the genus name that was historically employed for it, presenting this name between quotation marks.

Given the limitations and shortfalls of our data, which are solely based on mitochondrial DNA, we do not presume this updated generic arrangement the definitive scheme, but we intend to offer a working hypothesis that can be tested and formalized by further studies, as additional data become available. Careful taxonomic assessments with the inclusion of several lines of evidence, such as phenotypic information from sequenced and type material, are indispensable for this and other taxonomic issues of Sciurini to be properly addressed.

The first two major groups within Sciurini (A and B) compose the genera *Tamiasciurus* Trouessart, 1880 (including *T. douglasii* and *T. hudsonicus*) and *Rheithrosciurus* Gray, 1867 (including *R. macrotis*) as monophyletic groups, and we suggest the application of these names for the Groups A and B, respectively. The genus *Sciurus*, as broadly recognized in the past century—including Eurasian, Nearctic and Neotropical species (e.g. [1, 20–22])—is not monophyletic. This result has been also recovered in previous phylogenetic inferences with fewer species [7, 50–52] and is strongly supported by our analyses with denser taxon sampling. *Sciurus* can be restricted to the Eurasian clade, Group C, since it includes *vulgaris* Linnaeus, 1758, the type species of *Sciurus* Linnaeus, 1758. Other species included in the restricted concept of this genus are *S. anomalus* and *S. lis*. The remaining North American species are arranged in Groups D, E and F, for which four generic names are available, *Hesperosciurus*, *Otosciurus*, *Neosciurus* and *Parasciurus*. For Group D, two generic names were coined as subgenera by Nelson in 1899, *Otosciurus* for * aberti* and *Hesperosciurus* for * griseus*. Here we conservatively assign the oldest available genus-group name, *Hesperosciurus*, as a genus including *H. aberti* and *H. griseus*. Regarding Group E, the genus name *Parasciurus* Trouessart, 1880 is the only available one, and its type species is *niger* Linnaeus, 1758; therefore, we suggest the application of this name for this clade, composed of *P. nayaritensis*, *P. arizonensis*, *P. alleni* and *P. oculatus*, along with the type species. Finally, for Group F the only available name is *Neosciurus*, described by Trouessart, 1880 for *carolinensis* Gmelin, 1788, and this is the name that we tentatively apply to this lineage.

Group G, which comprises most Central American taxa, might be the most taxonomically conflicting group as it contains the type species of many genera, including *alfari* (type of *Microsciurus* J. A. Allen, 1895), *brochus* (type of *Syntheosciurus* Bangs, 1902) *deppei* (type of *Baiosciurus* Nelson, 1899) and *aureogaster* (the senior synonym of *Sciurus hypopyrrhus* Wagler, 1831, type species of *Echinosciurus* Trouessart, 1880) (Fig. 5). Allen [18] recognized five genera for the eight species or species-complex in this group. Subsequent authors recognized fewer genera, but none suggested a unique genus to contain those species. It is noteworthy that Moore [21] was the only author to anticipate a close relationship between *brochus* and *granatensis*, suggesting these taxa be placed under the genus *Syntheosciurus*. We partially follow the arrangement proposed by Allen [18] and Moore [21], and we suggest that the genus name *Microsciurus* should be applied for the clade formed by *alfari* and “species 1”; the name *Syntheosciurus* must be attributed to the group formed by *brochus* and *granatensis* (if Vivo and Carmignotto are correct [see below, on the discussion of the name of clade H] and *granatensis* is the type species of *Notosciurus*, this genus name is a junior synonym of *Syntheosciurus*); and we advocate the adoption of the name *Echinosciurus*, as the oldest available one, for the group formed by *aureogaster*, *colliaei*, *deppei*, *yucatanensis* and *variegatoides*.

Group H, composed of northwestern South American forms, includes five species traditionally allocated to the genus *Microsciurus* J. A. Allen, 1895 (*mimulus*, *similis*, *otinus*, *boquetensis*, and *isthmius*). However, the type species of the genus *Microsciurus* (*alfari* J. A. Allen, 1895), as demonstrated above, is nested within Group G and, therefore, the name *Microsciurus* cannot be applied to Group H. The other species recovered in this clade, *pucheranii* Fitzinger, 1867, is a controversial taxon. Allen [18] described the genus *Leptosciurus* and considered *pucheranii* as its type species. Moore [21] placed *pucheranii* in *Microsciurus*, and he was the only author to suggest a close relationship between this taxon and the small-sized species from the highlands of northwestern South America. Most other authors have allocated *pucheranii* to *Sciurus* (e.g. [1, 19, 20]), but Vivo and Carmignotto [2] included *pucheranii* and *granatensis* under the genus *Notosciurus* Allen, 1914. Their decision was based on the fact that: i) their concept of *N. granatensis* included *chysuros* Pucheran, 1845 as a subspecies (*N. g. chrysuros*) and *soederstroemi* Stone, 1914 as a junior-synonym of this subspecies; ii) Vivo and Carmignotto [2] followed Hershkovitz [53] who identified *N. rhoadsi* Allen, 1914 (the type species of *Notosciurus*) as a young specimen of *soederstroemi*. Therefore, for these authors, the name *Notosciurus* would be applied to *granatensis* (via its synonymy with *soederstromi*) and *pucheranii* (for their morphological similarity), and would have priority over the name *Leptosciurus*. However, our results did not recover *granatensis* and *pucheranii* as closely related taxa. Instead, *granatensis* was recovered in Group G, sister to *Syntheosciurus brochus*. Therefore, *Leptosciurus* seems to be the only available name for Group H, and we suggest the application of this name to the six species there nested.

Group I includes *stramineus* (type species of *Simosciurus* Allen, 1915) along with *nebouxii*. Vivo and Carmignotto [2] followed Allen [18] considering *Simosciurus* a valid genus, and we recover it as monophyletic based on mitogenomic data. Since *Simosciurus* is the only available name for this clade, which includes the type species of this genus, we believe it is the appropriate name for Group I. Our analyses also support the monophyly of the genus *Guerlinguetus* Gray, 1821, as recognized by both [2, 18], represented in our analyses by Group J. *Guerlinguetus* has been consistently employed for parts of this particular group of species, as full genus or subgenus, by several authors.

Described species recovered within Group K have been assigned to the genus *Microsciurus* by all authors. The unnamed lineage recovered within this Group (“species 2”) was also referred to the genus *Microsciurus* by [54, 55], but it has been referred to the genus *Syntheosciurus* by Vivo and Carmignotto [2]. Our data do not recover taxa of this group as closely related to the type species of *Microsciurus* or *Syntheosciurus*. Moreover, as the valid species in this group (*flaviventer* and *sabanillae*) were both described in genera currently occupied (*Macroxus* and *Microsciurus*, respectively), there seems to be no generic name available for Group K. Until more consistent morphologic dataset is available to allow a formal nomenclatural designation, we provisionally use the name “*Microsciurus*” for this clade (see Patton et al., 2015, for “*Handleyomys*”), as this was the name historically assigned to these species. An alternative measure would be to apply the genus name of the sister group (L) to this lineage, but we do not recommend this option as this would introduce more taxonomic confusion and instability.

Finally, Group L clustered species allocated in distinct genera according to [2, 18], or from a single but not monophyletic genus of Moore [21] and Thorington et al. [1]. At least five generic names have been applied to those species: *Notosciurus* Allen, 1914, *Leptosciurus* Allen, 1915, *Mesosciurus* Allen, 1915, *Hadrosciurus* Allen, 1915, and *Urosciurus* Allen, 1915. However, only *Hadrosciurus* and *Urosciurus* are possibly vacant here, and the correct assignment must be carefully evaluated in a comprehensive taxonomic study that includes a meticulous nomenclatural investigation for this group. However, in order to propose a tentative nomenclatural definition, as we have done for previous clades, we tentatively apply the name *Hadrosciurus* Allen, 1915, whose type species is *flammifer* Thomas, 1904, considered by Vivo and Carmignotto [2] as a junior-synonym of *igniventris* Wagner, 1842. This name was also advocated by Vivo and Carmignotto [2].

### Comments on species recognition and novelties

In this study, we sampled across the geographic ranges of several widespread taxa and, therefore, we were able to test the genetic integrity of currently recognized species of tree squirrels, especially those from South America. In contrast to our generic level analyses, most recognized species are highly supported as monophyletic groups in our analyses of mitochondrial genome data. Regarding Palearctic and Nearctic taxa, our sampling was remarkably inferior to the sampling for Neotropical taxa, with many species represented by as few as one or two individuals. As expected, all those species with more than one individual exhibit reciprocal monophyly in our phylogenomic analyses, following the species concepts presented by Thorington et al. [1].

Among the Central American taxa, the two cases of non-reciprocal monophyly were (i) the recovery of a sample identified as *richmondi* Nelson, 1898, from Nicaragua, nested within the clade associated with *Syntheosciurus granatensis*; and (ii) a specimen assigned to *venustulus* Goldman, 1912, from Panama, nested within the clade of *Microsciurus alfari*. Samples from *Syntheosciurus granatensis* compose two well-structured subclades, one of which includes specimens from the Ecuadorean and Peruvian Andes, Venezuela, and Trinidad and Tobago, and the other includes samples from the coast of Ecuador, Colombia, Nicaragua (referred to as *richmondi*), and Panama (Group G, Fig. 4a). Without the inclusion of additional specimens referred to *richmondi* and the careful examination of voucher material, we are unable to unveil, at this point, if this is a simple case of misidentification or if this taxon needs taxonomic re-evaluation. Our molecular species delimitation analyses provide distinct resolutions for the samples assigned to *granatensis* and *richmondi*, but none of them suggested the sample assigned to *richmondi* as a distinct species from the specimens of *granatensis*. Based on our phylogenetic results corroborated by BPP analysis, we recognize a single putative species, *Syntheosciurus granatensis*, for those samples. Regarding the second case, all samples of *Microsciurus alfari*, as well as the sample initially identified as *venustulus*, are from Panama and were suggested as a single species by all species delimitation analyses. Thus, we provisionally do not treat *venustulus* as a valid taxon until further evaluation with additional specimens.

Across South American lineages, our results indicate that *pucheranii* sensu [2] forms a non-monophyletic assemblage composed of two phylogenetically distant lineages included in Groups H and L. The concept of *pucheranii* adopted by Vivo and Carmignotto [2] includes specimens with a disjunct distribution, from the Central Andes of Colombia (assigned to *pucheranii pucheranii* Fitzinger, 1867) and from Peru and Brazil, Bolivia, and Argentina [assigned to three other subspecies named *pucheranii ignitus* Gray, 1867, *pucheranii boliviensis* Osgood, 1921, and *pucheranii argentinius* Thomas, 1921, respectively]. In our analyses, specimens of *pucheranii pucheranii* are recovered as part of Group H (Fig. 4b)—an Andean Trans-Andean clade composed of taxa from high elevation areas of northwestern South America. For this clade we provisionally apply the name *pucheranii* Fitzinger, 1867 to the species level, with the combination *Leptosciurus pucheranii*. Specimens associated with the remaining three subspecies were recovered as a clade nested within Group L (Fig. 4c), which includes Cis-Andean lowland taxa. For this lineage we suggest the application of the name *ignitus* Gray, 1867, as it has priority over *argentinius* and *boliviensis*, with the status of a full species. We did not intend to revalidate or describe new species in this contribution, however, as we were unable to use the current species concepts for the taxa mentioned above, we tentatively suggest this alternative arrangement, which is in accordance with the classification of Thorington et al. [1] at the species-group level; the name we propose is, thus, *Hadrosciurus ignitus*.

The concepts of *Guerlinguetus aestuans* and *G. brasiliensis* adopted by Vivo and Carmignotto [2] are also not monophyletic according to our analyses. Based on the geographic distribution of the samples, the subclades *G. aestuans* “a” and *G. aestuans* “b” include specimens associated with *Guerlinguetus aestuans*. The first subclade is composed of samples from Guyana and Venezuela, and the second of Brazilian samples from the southern bank of the Amazon river, west of the Tapajós river (Fig. 5d). The subclade *G. aestuans* “c” seems to encompass representatives of both *aestuans* and *brasiliensis*, since it includes specimens from north of the Amazon river (assigned to *aestuans* by those authors) and one specimen from Pernambuco, northern Atlantic Forest (assigned to *brasiliensis* by those authors). We referred to this last subclade as *G. aestuans* “c” as the great majority of samples within this lineage were previously assigned to *G. aestuans* and not to *G. brasiliensis*. The subclade *G. brasiliensis* is apparently composed of samples assigned exclusively to this nominal taxon, from southeastern Amazonia, eastern and southern Brazil. Therefore, we recognized specimens previously identified as *Guerlinguetus aestuans* and *G. brasiliensis* as composing four distinct lineages (see Fig. 4b), suggesting hidden diversity along the Amazon basin and implying an independently evolving lineage from the Gran Sabana and Mount Roraima, on the border of Brazil, Venezuela, and Guyana. This result was corroborated by most species delimitation analyses, except for one analysis (GMYC 2) in which *Guerlinguetus aestuans* “c” and *G. brasiliensis* were suggested as a unique putative species.

Our phylogenetic results also indicate the existence of three apparently unnamed lineages that might represent species to be described or revalidated, all of which were supported by molecular species delimitation methods. “Species 1” is represented by a specimen from Chocó, Colombia, which was previously identified as *Microsciurus mimulus*; however, this specimen was recovered as phylogenetically distant from other specimens of *M. mimulus* from Colombia and Ecuador (all of which clustered within Group H), and exhibited deep genetic divergence from its sister-taxa, *M. alfari* (see branch lengths on Fig. 4a). “Species 2” is represented in our analyses by five specimens from Peru (San Martin, Madre de Dios) and Brazil (Acre). Voucher material of this species, from San Martin, was analyzed by [54, 55]—who referred to it as *Microsciurus* sp.—and by [2]—who referred to it as *Syntheosciurus* sp. “Species 3” is represented by three specimens from two Amazonian lowland localities in Loreto (Peru), and is apparently sympatric with *Hadrosciurus spadiceus* at Rio Galvez, Nuevo San Juan. We did not find previous mention of this putative species in the literature.

Therefore, monophyletic groups representing currently recognized species in addition to the lineages representing putative unnamed taxa composed a set of 43 OTUs that we hypothesize as distinct species of tree squirrels. All South American OTUs were corroborated as unique species by at least two out of the three species delimitation analyses performed, except for one OTU, *Leptosciurus boquetensis*, which was only supported as a distinct species by BPP. Regarding non-South American taxa, our species delimitation analyses did not fully corroborate our working hypothesis. Discrepant results in the recognition of those species are likely a product of our sampling strategy, densely focused on South American taxa. At least BPP analyses are potentially affected by the number of samples per each presumed species, especially if using a dataset with few loci [56]. GMYC estimates might not be as affected by poorly represented species as BPP [57, 58], but can be strongly influenced by the way that the ultrametric tree is generated, which underprints the analysis [59]. Our results corroborate this assumption, as we found discrepant results suggesting 66 or 39 putative species using ultrametric trees generated with strict and relaxed molecular clocks, respectively.

Several studies have employed molecular species delimitation methods either as a standalone tool or as part of an integrative approach to delimit species [60–62]. Here, we advocate for the use of molecular species delimitation methods along with other sources of evidence, to avoid misleading species delimitation due to theoretical and/or methodological shortfalls (as exemplified above; see also [57–59]). Moreover, in many cases, when delimiting species based on a single-locus dataset, the estimates could be biased by the genealogical history of this locus which may or may not reflect the evolution of the group. As we used an exclusively mitogenomic dataset, we acknowledge that the evidence for pervasive natural selection, uniparental inheritance and the lack of recombination on the mitochondrial genome make it susceptible to evolutionary processes distinct to the nuclear genome [63, 64].

Considering the possible methodological weaknesses mentioned above and the shortfalls of our sampling of taxa and data, we evaluate the results of our molecular species delimitation analyses with special caution in some situations. For example, the genus *Tamiasciurus* was recently extensively revised through molecular analyses (including mitochondrial and nuclear genes) and ecological niche modeling, with over 250 specimens examined from throughout the distribution of the genus [24]. These authors found evidence for the recognition of *T. douglassi* and *T. hudsonicus* as valid species. Our analyses consistently failed to suggest these taxa, represented by a single sample each, as distinct species (see Fig. 4a). Another example is that some species delimitation analyses did not recognized *Sciurus lis* and *S. vulgaris* (GMYC 2) or *Sciurus lis*, *S. vulgaris* and *S. anomalus* (BPP) as distinct species. These taxa, which are represented in our dataset by only one terminal each, have been consistently recognized as distinct species based on molecular [25, 65] and karyotypical [27] data. They also exhibit consistent morphological differences in the number of pairs of mammae (a trait that seems not to be variable within species of tree squirrels [2]), which is three in *S. lis*, four *in S. vulgaris*, and five in *S. anomalus* (see Fig. 6).

These controversial results, especially for Eurasian and North American taxa, lead us to adopt a conservative posture that does not totally reject the hypotheses provided by the species delimitation analyses, but it is also in consonance with current taxonomic proposals based on wider sampling approaches (see examples above). For those cases of inconsistency regarding Eurasian, North American, and Central American taxa, and also for South American lineages where species complexes were suggested by one or two of the species delimitation analyses, subsequent investigations are certainly necessary. A thorough delimitation of species of Sciurini demands additional sampling for several taxa and, possibly, the inclusion of other lines of evidence such as phenotypic data and genetic data from independently evolving loci as in nuclear DNA.

### Phylogenetic and biogeographic remarks

Despite the discordances between our mitochondrial phylogenomic hypothesis and the taxonomic arrangements previously proposed for Nearctic and Neotropical tree squirrels, our results are biogeographically coherent, and consistent with most of the results obtained by the few molecular phylogenetic studies published for Sciurini, especially regarding the deepest nodes (major clades) within the tribe. Like Pečnerová and Martínková [66] and Pečnerová et al. [7], we recovered the genus *Tamiasciurus* as the first lineage to diverge within Sciurini, followed by *Rheithrosciurus* and *Sciurus*, although our study is the first to recover strong support for these relationships. Our results also corroborate the sister-taxa relationship between *Hesperosciurus griseus* and *H. aberti* found in those previous studies. The Central American clade obtained by [7] is similar in composition to our Group G, despite the different relationships within this group, recovered by us with strong support. In previous studies, the representativeness of South American taxa was very limited, and the relationships among the very few specimens were mostly discordant from our results. One relevant difference is that we recovered the Mexican endemics *Parasciurus alleni* and *P. oculatus* clustering with North American species, instead of within a South American clade as in [7].

Concerning the biogeographic pattern, we recovered two Palearctic clades (A and B), four Nearctic (C–F), and six Neotropical—one (G) predominantly composed of Central American with a few South American specimens included (all from Andean or Trans-Andean areas) and five (H–L) composed exclusively of South American taxa and Southern Panama specimens (Fig. 4). The distribution of those five predominantly South American clades seems to be defined by the Andean Cordillera. We found two clades occupying Andean and Trans-Andean areas (H and I) and three clades distributed on the Cis-Andean portion of the Continent (Groups J–L). Group H seems largely associated with montane habitats, while Group I is restricted to low elevation coastal areas near the sea-level. Regarding the Cis-Andean groups, Group J is the most widespread, occurring from the extreme east of South America, in the Atlantic Forest, to the Guiana Shield, and throughout the Amazon basin. The sister Groups K and L are largely sympatric and composed mostly by Amazonian lowland dwellers. In Group K, however, two lineages (“*Microsciurus” sabanillae* and “species 2”) reach mid-elevations on the east side of the Andean cordillera in Ecuador and Peru; and in Group L, one lineage (*Hadrosciurus ignitus*) is also found in high-altitude localities in Bolivia.

### Taxonomic consequence of the use of homoplastic traits in the study of tree squirrels

Historically, all genera proposed for Neotropical species of Sciurini were delimited based exclusively on morphological traits. For example, species of *Notosciurus* sensu [2] were diagnosed by the presence of three pairs of mammae and one upper premolar; and the genus *Microsciurus* sensu [1, 2, 18, 21] was defined, among other traits (e.g. small size), by the presence of three pairs of mammae and two upper premolars. Our results, however, indicate that these features are homoplastic, with similar conditions of both characters having evolved multiple times during the evolutionary history of tree squirrels. Morphologic convergence has been detected among several lineages of Sciuridae [67, 68] and, according to our data, seems to be common in both cranial and external traits of Sciurini. Grouping species based primarily on homoplastic characteristics might have led to some of the incongruences that we observe between the taxonomic arrangements and the molecular phylogeny recovered for tree squirrels. For instance, the genus *Microsciurus* sensu [1, 2, 18, 21] comprises a polyphyletic assemblage that clusters species sharing the same number of premolars and mammae. Phenotypic convergence has been previously detected for cranial traits in species formerly assigned to *Microsciurus* [7], and the use of homoplastic characters to diagnose this genus (e.g. by [2, 18]) can be claimed to explain the polyphyly of this taxon.

## Conclusions

The inclusion of historical samples was crucial to provide a comprehensive phylogenetic hypothesis for tree squirrels and to detect several taxonomic issues reported here. We investigated the different aspects that might have influenced the success of mitogenome recovery from historical samples of Sciurini and showed that the age of the specimen does not affect mitogenome completeness. This finding highlights the potential of old museum specimens—including holotypes and other taxonomically important material—for phylogenetic and evolutionary inferences. Our extensive sampling of museum specimens, allied with a modern next-generation sequencing approach, allowed us to recover the entire mitochondrial genome of several species of squirrels. After exploring our data by comparing the performance of matrices with alternative sampling strategies, we observed that the addition of a limited amount of missing data did not impact the estimated relationships nor caused significant change to the nodal support of the inferred phylogenies. The comparison of our results with proposed classification schemes illustrates that none of the taxonomic arrangements ever proposed fully corresponds to the phylogenetic structure recovered for Sciurini, with only a few of the currently recognized genera recovered as monophyletic. Therefore, we advance a preliminary and tentative nomenclatural designation for the taxa at the genus-group level, employing 13 names used in previous taxonomic classifications. Our phylogenetic reconstruction revealed that most recognized species are highly supported as monophyletic groups. Nevertheless, we found evidence supported by species delimitation analyses that the diversity of Neotropical tree squirrels is currently underestimated, with at least six lineages that might represent taxa to be named or revalidated. In summary, we hypothesize that the tribe Sciurini comprises 14 genera and 46 species (see Table 3)—of which 43 species were sampled here and three were not included in the present study, but we provisionally treated them as valid—, a more diverse estimate than recent catalogues [1, 2]. *Sciurus*, formerly the most diverse genus in the tribe, harbors only three species, while the genera *Leptosciurus* (with six species), *Hadrosciurus*, *Parasciurus* and *Echinosciurus* (all with five species each), are the most diverse within this radiation; the only monotypic genus is *Rheithrosciurus*. The Neotropical region harbors eight genera and 29 species. However, a detailed taxonomic investigation is necessary to carefully evaluate the applicability of the genus-level names, to provide diagnoses and or descriptions to them, as well as to evaluate the species-level taxonomy for those genera. Finally, by investigating the evolution of two morphological traits widely employed in the taxonomy of the group we revealed their homoplastic nature, helping to explain the incongruence between phylogenetic results and classificatory schemes presented so far.

**Table 3:** New taxonomic arrangement proposed for the tribe Sciurini based on the species analyzed and recognized as valid in this study. We did not analyze material from *Tamiasciurus fremonti* sensu [24], *Microsciurus santanderensis* and *M. simonsi* sensu [2], which are treated as valid but not listed below

**Family Sciuridae** G. Fischer, 1817	
**Subfamily Sciurinae** G. Fischer, 1817	
**Tribe Sciurini** G. Fischer, 1817	
**Genus*Tamiasiurus*** Trouessart, 1880	
*Tamiasciurus douglasii* (Bachman, 1839)	
*Tamiasciurus hudsonicus* (Erxleben, 1777)	
**Genus*Rheithrosciurus*** Gray, 1867	
*Rheithrosciurus macrotis* (Gray, 1856)	
**Genus*Sciurus*** Linnaeus, 1758	
*Sciurus anomalus* Gmelin, 1778	
*Sciurus lis* Temminck, 1844	
*Sciurus vulgaris* Linnaeus, 1758	
**Genus*Hesperosciurus*** Nelson, 1899	
*Hesperosciurus griseus* (Ord, 1818)	
*Hesperosciurus aberti* (Woodhouse, 1852)	
**Genus*Parasciurus*** Trouessart, 1880	
*Parasciurus arizonensis* (Coues, 1867)	
*Parasciurus nayaritensis* (J. A. Allen, 1890)	
*Parasciurus niger* (Linnaeus, 1758)	
*Parasciurus alleni* (Nelson, 1898)	
*Parasciurus oculatus* (Peters, 1863)	
**Genus*Neosciurus*** Trouessart, 1880	
*Neosciurus carolinensis* (Gmelin, 1788)	
**Genus*Microsciurus*** J. A. Allen, 1895	
*Microsciurus alfari* J. A. Allen, 1895	
*Microsciurus* “species 1”	
**Genus*Syntheosciurus*** Bangs, 1902	
*Syntheosciurus brochus* Bangs, 1902	
*Syntheosciurus granatensis* (Humboldt, 1811)	
**Genus*Echinosciurus*** Trouessart, 1880	
*Echinosciurus aureogaster* (F. Cuvier, 1829)	
*Echinosciurus colliaei* (Richardson, 1839)	
*Echinosciurus deppei* (Peters, 1863)	
*Echinosciurus yucatanensis* (J. A. Allen, 1877)	
*Echinosciurus variegatoides* (Ogilby, 1839)	
**Genus*Leptosciurus*** Allen, 1915	
*Leptosciurus mimulus* (Thomas, 1898)	
*Leptosciurus pucheranii* (Fitzinger, 1867)	
*Leptosciurus similis* (Nelson, 1899)	
*Leptosciurus otinus* (Thomas, 1901)	
*Leptosciurus boquetensis* (Nelson, 1903)	
*Leptosciurus isthmius* (Nelson, 1899)	
**Genus*Simosciurus*** J. A. Allen, 1915	
*Simosciurus nebouxii* (I. Geoffroy St.-Hilaire, 1855)	
*Simosciurus stramineus* (Gervais, 1841)	
**Genus*Guerlinguetus*** Gray, 1821	
*Guerlinguetus aestuans* “a”	
*Guerlinguetus aestuans* “b”	
*Guerlinguetus aestuans* “c”	
*Guerlinguetus brasiliensis* (Gmelin, 1788)	
**Genus*“Microsciurus”***	
*“Microsciurus” sabanillae* Anthony, 1922	
*“Microsciurus”* “species 2”	
*“Microsciurus” flaviventer* (Gray, 1867)	
**Genus*Hadrosciurus*** J. A. Allen, 1915	
*Hadrosciurus* “species 3”	
*Hadrosciurus igniventris* (Wagner, 1842)	
*Hadrosciurus pyrrhinus* (Thomas, 1898)	
*Hadrosciurus ignitus* (Gray, 1867)	
*Hadrosciurus spadiceus* (Olfers, 1818)	

## Methods

### Sampling

In order to obtain a thorough sampling of Sciurini, we gathered a total of 271 samples from 27 scientific collections (Additional file 5), including 177 modern samples (ethanol-preserved tissue) and 94 historical samples (obtained from dry museum specimens). Historical samples were collected with the specific purpose of complementing missing taxa or important geographic variants, with special effort on Neotropical taxa. When collecting tissues from dry museum specimens, we prioritized sampling remains of muscular tissue adherent to skulls (“osteocrusts”) or, if those were not available, we obtained skin clips. Sampling from dry museum specimens followed strict procedures, including changing gloves and cleaning all instruments and working surfaces with 15% bleach followed by sterilized water between each sample (see detailed protocol in [12]).

The sampled material includes 40 out of the 43 currently recognized species of Sciurini (sensu [1, 2]). The unsampled taxa include *Microsciurus santanderensis* (known from few specimens collected between the Río Magdalena and the western slopes of the Cordillera Oriental in Colombia [69];), *Microsciurus simonsi* (known from few localities west of the Andes, in the Ecuadorian provinces of Bolívar and Pichincha [2]), and *Tamiasciurus fremonti*, revalidated from the synonymy of *T. hudsonicus* by Hope et al. [24] (known from the southwestern United States in the southern Rockies, Sacramento Mountains in New Mexico, and the southwestern Sky Islands [24, 70]). Additionally, we sampled three species of the tribe Pteromyini (sister to Sciurini [50]) to be used as outgroups. A complete list of the 232 specimens used in our analyses (for which we recovered at least 20% of the mitogenome) indicating the GenBank accession numbers and accompanied by geographic data and other relevant information is provided as Additional file 6.

### Taxonomic identifications

Specimens of Sciurini were identified at the species level following the latest taxonomic hypotheses available for each taxon. Samples of the North American genus *Tamiasciurus* were identified following Hope at al. [24]. South American material was identified following Vivo and Carmignotto [2], as well as the Central American taxa included on the taxonomic hypothesis of those authors (assigned by them to the genus *Microsciurus*: *alfari*, *boquetensis* and * venustulus*). For the remaining Central American taxa (not included on the taxonomic hypothesis of [2]), North American (except by *Tamiasciurus*), and Eurasian taxa, we have identified specimens following Thorington et al. [1].

For several specimens, especially those housed at the American Museum of Natural History (AMNH) and at the Smithsonian National Museum of Natural History (USNM), we kept the museum identifications, which had been made by some of the main authorities on tree squirrel taxonomy (e.g. R. W. Thorington and M. de Vivo). For material not previously identified, we were able to perform the identifications by examining the morphology of the vouchers, consulting original descriptions and other relevant literature. In cases for which we were not able to examine vouchers, we accepted original museum identifications if i) those identifications correspond to the known geographic distribution of the taxon in question, and ii) phylogenetic analyses of their DNA sequences were consistent with the museum identification.

### DNA extractions

DNA of historical samples was extracted in an isolated ancient DNA facility at the Smithsonian’s Center for Conservation Genomics (CCG), using a standard phenol-chloroform protocol (see detailed protocol in [12]). The ancient DNA lab at CCG is physically separated from the main laboratory, and no fresh tissue/DNA samples or PCR amplifications are allowed, to minimize and control sample contamination. Extractions included a long lysis step, between 3 to 5 days. Each batch of historical sample extraction included from seven to 11 specimens and a negative control to monitor for contamination. DNA extractions of modern tissues were performed in the main laboratory at CCG using the DNeasy® Blood & Tissue kit, following manufacture’s protocol (Qiagen Inc.), with an overnight lysis step. Total DNA concentrations were measured using a Qubit 2.0 fluorometer (Thermo Fisher Scientific).

### Library preparations and mtDNA amplification

For historical samples, an initial amount of 33 μl of DNA (regardless of concentration) was purified and concentrated using 5x SPRI magnetic beads [71]. DNA extracted from preserved tissues was sonicated to randomly shear with QSonica Q800R, using 25% of amplitude and 5 min of on/off pulse. Sheared DNA was visualized on agarose gel to confirm the resulting fragment size around 300 bp. Approximately 500 ng of sheared modern DNA was then purified using 5x SPRI magnetic beads [71].

Library preparations were performed using the KAPA LTP Library Preparation Kit (Roche Sequencing) following the manufacturer’s protocol. Subsequently, Nextera-style indices and KAPA HiFi Hotstart ReadyMix (Roche Sequencing) were used for indexing PCRs (iPCR). The iPCR profile included an initial denaturation at 98 °C for 45 s, a final extension at 72 °C for 7 min, and 14 (for modern samples) or 16 to 18 (for historical samples) cycles of amplification, with denaturation at 98 °C for 15 s, annealing at 60 °C for 30 s and extension at 72 °C for 60 s. The iPCR products were purified using 1.8x SPRI magnetic beads, quantified with a Qubit 2.0 fluorometer (Thermo Fisher Scientific) and visualized on a 1.5% agarose gel.

Libraries were multiplexed in equimolar ratios for target capture and enrichment of Ultraconserved Elements (UCEs) using similar procedures as described in [34]. We did not perform capture or enrichment of the mtDNA; the mitogenomes were obtained as a byproduct of the UCE enrichment without the need of an extra step for mitochondrial-specific enrichment or amplifications. For historical samples we pooled up to four libraries and for modern samples up to eight libraries. No historical samples were pooled with modern samples to avoid biased enrichment. Post-capture amplifications were performed using KAPA HiFi Hotstart ReadyMix (Roche Sequencing), with the following profile: initial denaturation at 98 °C for 2 min, a final extension at 72 °C for 7 min, and 15 (for modern samples) or 16 (for historical samples) cycles of amplification, with denaturation at 98 °C for 20 s, annealing at 60 °C for 30 s, and extension at 72 °C for 30 s. A 1.8x SPRI magnetic bead cleanup was performed subsequently.

### Quantification and sequencing

Cleaned amplifications were quantified using a Qubit 2.0 fluorometer (Thermo Fisher Scientific) and visualized on a Bioanalyzer (Agilent) with high sensitivity kits. Equimolar pooling of samples for sequencing was based on the concentration (ng/μl) and on the average size (bp) of DNA fragments. High concentration of dimmers was common, especially for historical samples. This problem was solved by size-selecting the fragments of DNA between 200 and 550 bp using a Pippin Prep (Sage Science). Both size-selection and sequencing were performed at the DNA Sequencing Center at the Brigham Young University, Utah, and at the Vincent J. Coates Genomics Sequencing Laboratory at the University of California, Berkeley. Illumina sequencing was done on a Hi-Seq 2500 125 PE and on a Hi-Seq 4000 150 PE using the Illumina Free Adapter Blocking Reagent to prevent index hopping.

### Data processing

Raw FASTQ files were provided by the sequencing cores. The raw data was processed to extract mtDNA as “off-target sequences” of the UCE capture [34]. Raw reads were cleaned for removal of adapter contamination and low-quality bases using Illumiprocessor 2.0 [72, 73]. Partial and complete mitochondrial genomes were recovered using Geneious R11 [74]. Clean reads (paired P1 and P2, plus singletons) were incorporated in Geneious and mapped to a reference mitochondrial genome (*Sciurus vulgaris* available in GenBank with accession number AJ238588) using the following mapping parameters: a minimum map quality of 30—which means that with 99.9% confidence the mapping is correct; a minimum overlap of 25 base-pairs for a read to be assembled into a contig; a minimum overlap identity of 85% (i.e. the minimum percentage of bases that must be identical in the overlapping region for a read to be assembled) with maximum of 15% of mismatches per read; a maximum of 10% of gap per read, with maximum gap size of 10 base-pairs. Up to five iterative mapping cycles were performed to find the greatest number of matching reads. Consensus sequences were generated with a minimum coverage of 3x. The mitochondrial genomes assembled were visually inspected and the coding genes were translated. We submitted all complete mitogenomes recovered to be annotated by MITOS [75] and the remaining partial genomes obtained were manually annotated based on the annotations provided by MITOS. All annotations were manually added to the sequences using Geneious R11 [74], where we performed visual inspection to certify that the beginning and end of the annotated coding sequences (CDS) matched with the translations of start and stop codons. We converted the mitogenome annotation of one species of tree squirrel (*Guerlinguetus brasiliensis*) into a graphical map using OGDRAW 1.3.1 [76], to exemplify the genome synteny in the group (Fig. 2).

### Sequence alignment and dataset composition

The consensus mitochondrial genomes were aligned using MUSCLE [77] with up to eight interactions. In order to examine the possible effects of including and excluding characters and taxa with missing data on our phylogenetic inferences, we generated five datasets considering distinct percentages of mitogenome completeness per sample: Dataset 1 included only specimens for which we obtained full mitochondrial genomes (92 specimens with no missing data); Dataset 2 included samples for which at least 80% of the mitogenome was recovered (162 specimens with < 20% of missing data per sample); Dataset 3 included samples for which at least 60% of the mitogenome was recovered (186 specimens with < 40% of missing data per sample); Dataset 4 included samples for which a minimum of 40% of the mitogenome was recovered (210 specimens with < 60% of missing data per sample); and Dataset 5 included samples for which a minimum of 20% of the mitogenome was recovered (232 specimens with < 80% of missing data per sample). We did not include the 39 samples for which we recovered less than 20% of the mitogenome in our datasets (Table 1). Therefore, from the 271 samples that we attempted to sequence, we only used a total of 232 samples in our analyses.

### Mitochondrial genome recovery for historical museum samples

To investigate how different factors might influence the success of mitochondrial genome recovery for historical samples, we compared the completeness of mitogenome obtained with tissue type (osteocrusts versus skin clips), and museum location (samples from scientific collections in NA versus SA) using Pearson’s Chi-squared test. We also investigated the relationship between collection year and mitogenome recovery using a linear regression model. For the last, we only included osteocursts (which compose the great majority of our historical samples) from NA museums (our main source of historical samples). This was done to avoid bias related to tissue type and storage conditions (NA museums have similar storage conditions and standardized procedures to preserve specimens, while storage conditions and procedures are highly heterogeneous in SA collections). All analyses were performed in RStudio 1.1.463 (RStudio, Inc.).

### Phylogenetic analyses

Phylogenetic analyses were performed for each one of our five datasets using Maximum Likelihood (ML) in RaxML 8.2.7 [78]. Ten independent searches were performed under the GTR + G nucleotide substitution model (RAxML only implements GTR-based models of nucleotide substitution). The best-scoring ML trees were selected to draw the bootstrap support values obtained by running 1000 replicates using the “thorough standard bootstrap” optimization option. In addition to the ML analyses performed on all datasets, Bayesian inference (BI) in MrBayes 3.2.6 [79] was performed exclusively on Dataset 5 (see below “Phylogenetic inferences and the effect of missing data” for details). For the BI, the best-fit partitioning scheme and models of nucleotide substitution were specified as determined by PartitionFinder [80] under the corrected Akaike Information Criterion (AIC). We defined 39 separate data blocks in our alignment: 22 transfer RNAs (tRNAs), 13 protein-coding genes (PCGs), two ribosomal RNAs (rRNAs), one origin of replication, and a control region (D-loop). The PCGs were also separated by codon positions. Therefore, our search for partitioning schemes and models occurred independently in 65 data blocks of the mitochondrial genome. The BI was performed in parallel [81] with two independent runs and with four chains each. The MCMC (Markov Chain Monte Carlo) simulations proceeded for 4 X 10^7^ generations, sampling every 4000 generations. Nodal support was obtained as posterior probability. The quality and convergence of MCMC runs were verified in Tracer 1.7 [82]. Topologies from both ML and BI analyses were edited in FigTree 1.4.4 (http://tree.bio.ed.ac.uk/software/figtree/). Phylogenetic analyses were conducted on the Smithsonian Institution High Performance Cluster (SI/HPC; 10.25572/SIHPC).

### Species delimitation analyses

In order to provide a quantitative test regarding species limits in Sciurini, we applied two species delimitation methods that are amongst the most widely used in the recent literature: the generalized mixed Yule-coalescent model (GMYC [83]), and the Bayesian multispecies coalescent approach in the software Bayesian Phylogenetics and Phylogeography (BPP [84]).

GMYC was initially developed for analyses of single-locus data, however, it has been frequently applied to concatenated matrix of multilocus data by assuming a common genealogical history [56]. This method aims to identify the limit between a Yule speciation process and the intraspecific coalescence using a likelihood approach and a provided ultrametric tree [83]. We used BEAST 2.6.1 [85] to obtain ultrametric trees. We generated two trees using a concatenated matrix of the 13 PCGs, one applying a strict molecular clock and another with a relaxed log-normal clock, both with a Yule tree prior. BEAST runs were conducted by 100 million generations of Markov chain Monte Carlo (MCMC), sampling every 20,000 generations. The results of MCMC runs were inspected in Tracer 1.7 [82] to confirm a minimum of 200 effective sample size for all parameters. Ultrametric trees were summarized with TreeAnnotator v2.6.0 [85], considering 10% of burnin and selecting the maximum clade credibility as the target tree. We performed GMYC analyses using the strict molecular clock generated tree (GMYC 1) and using the relaxed log-normal clock generated tree (GMYC 2), to verify how this change may affect species delimitation. Both GMYC analyses were performed using the R package splits v1.0–19 [86] and we selected the single-threshold version of GMYC, as it has been shown to outperform the multiple-threshold version [83, 87].

Different from GMYC, BBP was designed to test models of evolution on multilocus datasets. BPP is a Bayesian MCMC based program for delimiting species under the multispecies coalescent model [84]. We used BPP v4.1.4 [88] to analyze a multilocus dataset composed of the 13 mitochondrial PCGs. We provided a guided species-tree based on our ML analysis of Dataset 5, considering as terminals (species to be tested) the 43 OTUs recognized through the morphological identifications and confirmed as monophyletic groups by our phylogenetic inferences. We performed multiple BPP analyses with varying priors of ancestral population size (θ) and root age (τ). Our exploratory analyses showed that only assuming large ancestral population sizes (θ = 0.2) and deep divergences among species (τ = 0.2) we were able to reach convergence between MCMC runs and to obtain satisfactory effective sample sizes (near or above 200) for the analytical parameters. Each BPP analysis was conducted by 300,000 generations of MCMC and 10% of the samples were discarded as burn-in. Convergence on the estimates were verified in Tracer 1.7 [82].

### Morphological character evolution

We performed ancestral state reconstructions (ASR) of two discrete traits: “number of upper premolars” and “number of pairs of mammae” using a ML approach. These morphological characters were selected considering their broad use in taxonomic studies of Neotropical squirrels [2, 18, 21], and they are representatives of both cranial and external features that have been traditionally employed to assign species to genera and to establish genera limits. To reconstruct the evolution of number of upper premolars, we recognized the states *one premolar*, and *two premolars*. To reconstruct the evolution of the number of pairs of mammae, we assigned species to either *three pairs*, *four pairs*, or *five pairs* of mammae. To score species, we compiled information from literature and examined specimens housed at the American Museum of Natural History (AMNH) and National Museum of Natural History (USNM), including all species included in our phylogenetic hypothesis. State coding for all species and relevant scoring details are provided as Additional file 7.

Reconstructions were inferred in RStudio 1.1.463 (RStudio, Inc.), on an ML tree derived from our Dataset 5 trimmed to the species level. We tested three models of evolution using the function “fitDiscrete” from package geiger [89]: Markov model with equal rates (Mk-ER); Markov model with symmetric rates (Mk-SYM); and Markov model with all rates different (Mk-ARD). The best models, selected with base on their support from a vector of AIC scores, were then used to perform the ancestral character estimation using the function “ace” from package ape [90]. The results were displayed on the ML tree using the function “plot.phylo” from ape [90].

## Supplementary information

**Additional file 1. **Mitogenome recovery success (completeness) obtained from historical samples according to tissue type and museum location. (a) Tissue type: osteocrust samples (*N* = 70, X = 52.7%) and skin clips (*N* = 10, X = 37.6%). (b) Location of scientific collection: North America (*N* = 66, X = 55.9%) and South America (*N* = 14, X = 26.7%). Samples from both NA and SA collections are included on the tissue type comparison, while both osteocrusties and skin clips are included on the museum location comparison.

**Additional file 2.** Best-fitting models of sequence evolution used on BI analyses. Numbers between brackets are codon positions.

**Additional file 3.** Summary of models tested to reconstruct the evolution of number of premolars, with respective AIC scores, delta values and AIC weights.

**Additional file 4.** Summary of models tested to reconstruct the evolution of pairs of mammae, with respective AIC scores, delta values and AIC weights.

**Additional file 5.** Catalog data of voucher material.

**Additional file 6.** List of specimens successfully sequenced and analyzed in this study, with geographic information and GenBank accession numbers for complete mitochondrial genomes. Voucher numbers in bold refer to specimens from which we have used dried tissue (instead of ethanol-preserved tissue). Taxonomic identifications follow the new arrangement proposed here (see text for detailed explanation). The column “Group” refers to the major groups recognized within Sciurini, as recovered by our analyses (see Figs. 3 and 4). See Catalog data of voucher material (Additional file 5) for explanations of voucher acronyms.

**Additional file 7.** State coding used on ancestral state reconstruction analyses. Number of upper premolars were coded as one (1) or two (2), and number of pairs of mammae were coded as three (3), four (4), or five (5). Taxonomic identifications follow the new arrangement proposed here (see text for detailed explanation).

## Data Availability

The datasets generated and analyzed during the current study are available in the Dryad Digital Repository (10.5061/dryad.9w0vt4bc9). GenBank accession numbers are provided for all complete mitochondrial genomes in the Additional file 6.

## Abbreviations

AIC: Akaike information criterion
AICw: Akaike information criterion weight
AMNH: American Museum of Natural History
ASR: Ancestral state reconstructions
BI: Bayesian inference
BPP: Bayesian phylogenetics and phylogeography
CA: Central America
CCG: Smithsonian’s Center for Conservation Genomics
GMYC: Generalized mixed Yule-coalescent model
MCMC: Markov Chain Monte Carlo
Mk-ARD: Markov model with all rates different
Mk-ER: Markov model with equal rates
Mk-SYM: Markov model with symmetric rates
ML: Maximum Likelihood
MRCA: Most recent common ancestor
NA: North America
OUT: Operational Taxonomic Units
PCGs: Protein-coding genes
SA: South America
USNM: National Museum of Natural History
