# Supplementary material for: Museomics of tree squirrels: a dense taxon sampling of mitogenomes reveals hidden diversity, phenotypic convergence, and the need of a taxonomic overhaul
Source: BMC Evol Biol. 2020 Jun 26;20:77. doi: 10.1186/s12862-020-01639-y (PMC7320592; doi:10.1186/s12862-020-01639-y)
Supplement: Supplementary file 1 — Additional file 1. Mitogenome recovery success (completeness) obtained from historical samples according to tissue type and museum location. (a) Tissue type: osteocrust samples (N = 70, X = 52.7%) and skin clips (N = 10, X = 37.6%). (b) Location of scientific collection: North America (N = 66, X = 55.9%) and South America (N = 14, X = 26.7%). Samples from both NA and SA collections are included on the tissue type comparison, while both osteocrusties and skin clips are included on the museum location comparison. [file 12862_2020_1639_MOESM1_ESM.pdf]

Additional file 1

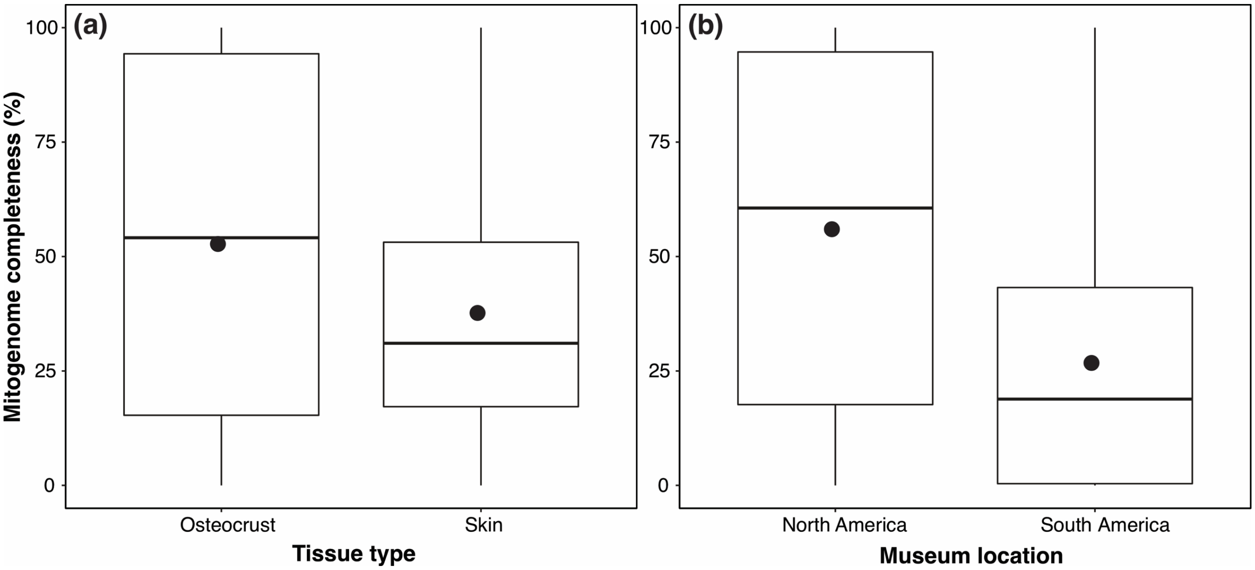

Mitogenome recovery success (completeness) obtained from historical samples according to tissue type and museum location. (a) Tissue type: osteocrust samples (N = 70, X = 52.7%) and skin clips (N = 10, X = 37.6%). (b) Location of scientific collection: North America (N = 66, X = 55.9%) and South America (N = 14, X = 26.7%). Samples from both North America and South America collections are included on the tissue type comparison, while both osteocrusties and skin clips are included on the museum location comparison.
