# Supplementary material for: Museomics of tree squirrels: a dense taxon sampling of mitogenomes reveals hidden diversity, phenotypic convergence, and the need of a taxonomic overhaul
Source: BMC Evol Biol. 2020 Jun 26;20:77. doi: 10.1186/s12862-020-01639-y (PMC7320592; doi:10.1186/s12862-020-01639-y)
Supplement: Supplementary file 2 — Additional file 2. Best-fitting models of sequence evolution used on BI analyses. Numbers between brackets are codon positions. [file 12862_2020_1639_MOESM2_ESM.pdf]

Additional file 2

Best-fitting models of sequence evolution used on BI analyses. Numbers between brackets are codon positions.

Subset	Best Model	Number of sites	Mitogenome Partitions
1	GTR+I+G	4578	CDS-ATP8 (2), tRNA-Glu, CDS-CYTB (1), CDS-NADH1 (1), rRNA-12S, tRNA-Ala, CDS-NADH3 (1), CDS-NADH5 (1), CDS-NADH6 (2), tRNA-Cys, tRNA-Asp, tRNA-Trp, CDS-NADH6 (1), tRNA-Ser-AGY, tRNA-Arg, tRNA-Leu-UUR, tRNA-Asn, tRNA-Pro, CDS-NADH4 (3), CDS-NADH2 (1), tRNA-Ser-UCN, tRNA-Phe, CDS-ATP8 (1), tRNA_Lys
2	SYM+I+G	1218	CDS-COII (1), CDS-COI (1), tRNA-Tyr, CDS-COIII (1), tRNA-Val, tRNA-Gly
3	GTR+I+G	1779	tRNA-lle, tRNA-Thr, rRNA-16S, tRNA-His
4	GTR+I+G	4095	CDS-COII (2), CDS-COI (2), CDS-NADH1 (2), CDS-NADH5 (2), CDS-NADH4 (1), CDS-ATP6 (3), CDS-NADH4L (1), CDS-ATP6 (1), CDS-CYTB (2), CDS-COIII (2), tRNA-Leu-CUN, CDS-NADH2 (2), tRNA-Gln, rep-origin, tRNA-Met, CDS-NADH4L (2), CDS-NADH3 (2)
5	GTR+I+G	3708	CDS-COIII (3), CDS-COI (3), CDS-NADH4 (2), CDS-CYTB (3), CDS-NADH6 (3), CDS-NADH2 (3), CDS-NADH4L (3), CDS-NADH3 (3), CDS-NADH5 (3), CDS-NADH1 (3), CDS-COII (3), CDS-ATP6 (2)
6	GTR+I+G	1133	D-loop, CDS-ATP8 (3)
