# Supplementary material for: Museomics of tree squirrels: a dense taxon sampling of mitogenomes reveals hidden diversity, phenotypic convergence, and the need of a taxonomic overhaul
Source: BMC Evol Biol. 2020 Jun 26;20:77. doi: 10.1186/s12862-020-01639-y (PMC7320592; doi:10.1186/s12862-020-01639-y)
Supplement: Supplementary file 3 — Additional file 3. Summary of models tested to reconstruct the evolution of number of premolars, with respective AIC scores, delta values and AIC weights. [file 12862_2020_1639_MOESM3_ESM.pdf]

Additional file 3

Summary of models tested to reconstruct the evolution of number of premolars, with respective AIC scores, delta values and AIC weights.

Model	AIC	Delta	AICw
Mk1-ER	42.872	0.000	0.686
Mk-ARD	44.435	1.563	0.314
