# Supplementary material for: Museomics of tree squirrels: a dense taxon sampling of mitogenomes reveals hidden diversity, phenotypic convergence, and the need of a taxonomic overhaul
Source: BMC Evol Biol. 2020 Jun 26;20:77. doi: 10.1186/s12862-020-01639-y (PMC7320592; doi:10.1186/s12862-020-01639-y)
Supplement: Supplementary file 4 — Additional file 4. Summary of models tested to reconstruct the evolution of pairs of mammae, with respective AIC scores, delta values and AIC weights. [file 12862_2020_1639_MOESM4_ESM.pdf]

**Additional file 4**

Summary of models tested to reconstruct the evolution of pairs of mammae, with respective AIC scores, delta values and AIC weights.

| Model | AIC | Delta | AICw |
|---|---|---|---|
| Mk1-ER | 65.321 | 3.599 | 0.126 |
| Mk-SYM | 61.722 | 0.000 | 0.763 |
| Mk-ARD | 65.581 | 3.859 | 0.111 |
