# Supplementary material for: Museomics of tree squirrels: a dense taxon sampling of mitogenomes reveals hidden diversity, phenotypic convergence, and the need of a taxonomic overhaul
Source: BMC Evol Biol. 2020 Jun 26;20:77. doi: 10.1186/s12862-020-01639-y (PMC7320592; doi:10.1186/s12862-020-01639-y)
Supplement: Supplementary file 5 — Additional file 5. Catalog data of voucher material. [file 12862_2020_1639_MOESM5_ESM.pdf]

Additional file 5

Catalog data of voucher material.

Voucher material for genetic data analyzed in this study (see Additional file 6) is preserved in the following scientific collections: American Museum of Natural History, USA (AMNH); Colección Boliviana de Fauna, Bolivia (CBF); Coleção de Mamíferos “Alexandre Rodrigues Ferreira”, Universidade Estadual de Santa Cruz, Brazil (CMARF); Field Museum of Natural History, USA (FMNH); Instituto de Desenvolvimento Sustentável Mamirauá, Brazil (IDSM); Coleções Científicas Fauna do Amapá, Instituto de Pesquisas Científicas e Tecnológicas do Estado do Amapá, Brazil (IEPA); Institut des Sciences de l’Évolution Montpellier, France (ISEM); University of Kansas Natural History Museum, USA (KU); Coleção do Laboratório de Mamíferos, Escola Superior de Agricultura “Luiz de Queiroz”, Universidade de São Paulo, Brazil (LMUSP); Louisiana Museum of Natural History, USA (LSUMZ); Museu de Ciências Naturais, Fundação Zoobotânica do Rio Grande do Sul, Brazil (MCN-FZB); Coleção de Mastozoologia do Museu de Ciências Naturais, Pontifícia Universidade Católica de Minas Gerais, Brazil (MCN-M); Museu de Ciências Naturais, Universidade Luterana do Brasil, Brazil (MCNU); Museu Nacional da Universidade Federal do Rio de Janeiro, Brazil (MN); Museu Paraense Emilio Goeldi, Brazil (MPEG); Museum of Southwestern Biology, University of New Mexico, USA (MSB); Coleção de Tecidos de Vertebrados, Departamento de Zoologia, Instituto de Biociências, Universidade de São Paulo, Brazil (MTR); Museo de Historia Natural, Universidad Nacional Mayor de San Marcos, Peru (MUSM); Museum of Vertebrate Zoology, University of Califórnia, USA (MVZ); Museu de Zoologia da Universidade de São Paulo, Brazil (MZUSP); Sam Noble Oklahoma Museum of

Natural History, USA (OMNH); Museum of Texas Tech University, USA (TTU); Coleção de Tecidos Animais, Universidade Federal do Espírito Santo, Brazil (UFES-CTA); Coleção Zoológica, Instituto de Biociências, Universidade Federal de Mato Grosso, Brazil (UFMT); Universidade Federal do Pará, Brazil (UFPA); Universidade Federal de Santa Catarina, Brazil (UFSC); Smithsonian National Museum of Natural History, USA (USNM).

Uncatalogued vouchers are identified (in Additional file 6) by the acronym of the museum where they are currently housed followed by field numbers (in parentheses) with the following prefixes: ANRA = A. Ravetta (MPEG); EFA = E. F. Abreu-Jr. (LMUSP); GTG = G. T. Garbino (MZUSP); and LHE = L. H. Emmons (CBF). The prefixes BM, DTM, ENM, ICA, JAP, and MJ correspond to field series at the LMUSP; CN, DICO, PECC, RDSC, and RETA correspond to field series at the MPEG; AUATI correspond to field series at the IDSM; LAB correspond to field series at the UFMT; and JMIJ correspond to field series at the UFPA.
