# Supplementary material for: Museomics of tree squirrels: a dense taxon sampling of mitogenomes reveals hidden diversity, phenotypic convergence, and the need of a taxonomic overhaul
Source: BMC Evol Biol. 2020 Jun 26;20:77. doi: 10.1186/s12862-020-01639-y (PMC7320592; doi:10.1186/s12862-020-01639-y)
Supplement: Supplementary file 6 — Additional file 6. List of specimens successfully sequenced and analyzed in this study, with geographic information and GenBank accession numbers for complete mitochondrial genomes. Voucher numbers in bold refer to specimens from which we have used dried tissue (instead of ethanol-preserved tissue). Taxonomic identifications follow the new arrangement proposed here (see text for detailed explanation). The column “Group” refers to the major groups recognized within Sciurini, as recovered by our analyses (see Figs. 3 and 4). See Catalog data of voucher material (Additional file 5) for explanations of voucher acronyms. [file 12862_2020_1639_MOESM6_ESM.pdf]

Additional file 6

List of specimens successfully sequenced and analyzed in this study, with geographic information and GenBank accession numbers for complete mitochondrial genomes. Voucher numbers in bold refer to specimens from which we have used dried tissue (instead of ethanol-preserved tissue). Taxonomic identifications follow the new arrangement proposed here (see text for detailed explanation). The column "Group" refers to the major groups recognized within Sciurini, as recovered by our analyses (see Figures 3 and 4). See Catalog data of voucher material (Additional file 5) for explanations of voucher acronyms.

Voucher	Species	Group	Country	State/ Department	Municipality/Locality	Latitude	Longitude	Accession mitogenome
KU 144565	*"Microsciurus"* "species 2"	K	Peru	Madre de Dios	Reserva Cuzco Amazonico, 14 km E of Puerto Maldonado	-12.600000	-69.054490	
**MUSM 7922**	*"Microsciurus"* "species 2"	K	Peru	San Martín	Pataz, Parque Nacional del Río Abiseo, Río El Susto ca. 30 km NE Pataz	-7.647143	-77.417009	
**MUSM 7971**	*"Microsciurus"* "species 2"	K	Peru	San Martín	Pataz, Parque Nacional del Río Abiseo, Vilcabamba del Pajate	-7.647143	-77.417009	
MVZ 193716	*"Microsciurus"* "species 2"	K	Brazil	Acre	Ocidente, right bank Rio Juruá	-8.566667	-72.800000	
MVZ 193718	*"Microsciurus"* "species 2"	K	Brazil	Acre	Ocidente, right bank Rio Juruá	-8.566667	-72.800000	
AMNH 272819	*"Microsciurus" flaviventer*	K	Peru	Loreto	Rio Galvez, Nuevo San Juan	-5.250000	-73.166670	MT259112
FMNH 170300	*"Microsciurus" flaviventer*	K	Peru	Madre de Dios	Quebrada Aguas Calientes, left bank, Rio Alto Madre de Dios, 2.75 km E Shintuya	-12.668330	-71.269000	
FMNH 170301	*"Microsciurus" flaviventer*	K	Peru	Madre de Dios	Quebrada Aguas Calientes, left bank, Rio Alto Madre de Dios, 2.75 km E Shintuya	-12.668330	-71.269000	
FMNH 170302	*"Microsciurus" flaviventer*	K	Peru	Madre de Dios	Quebrada Aguas Calientes, left bank, Rio Alto Madre de Dios, 2.75 km E Shintuya	-12.668330	-71.269000	
MPEG 41774	*"Microsciurus" flaviventer*	K	Brazil	Amazonas	Jutai, RDS Cujubim	-4.935490	-68.173360	MT259116
MSB 57066	*"Microsciurus" flaviventer*	K	Bolivia	Pando	Santa Rosa	-12.216670	-68.400000	
MUSM 13307	*"Microsciurus" flaviventer*	K	Peru	Loreto	Rio Galvez, Nuevo San Juan	-5.250000	-73.166670	MT259117
MVZ 190348	*"Microsciurus" flaviventer*	K	Brazil	Amazonas	Barro Vermelho, left bank Rio Juruá	-6.466667	-68.766667	
MVZ 191205	*"Microsciurus" flaviventer*	K	Brazil	Amazonas	Barro Vermelho, left bank Rio Juruá	-6.466667	-68.766667	MT259120

MVZ 193714	*"Microsciurus" flaviventer*	K	Brazil	Acre	Flora [=Fazenda Santa Fé], left bank Rio Juruá	-8.600000	-72.850000	MT259121
MVZ 193715	*"Microsciurus" flaviventer*	K	Brazil	Acre	Ocidente, right bank Rio Juruá	-8.566667	-72.800000	MT259122
KU 158247	*"Microsciurus" flaviventer*	K	Peru	Loreto	Teniente Lopez	-2.583330	-76.116670	
KU 158251	*"Microsciurus" flaviventer*	K	Peru	Loreto	Teniente Lopez, 1.5 km N of	-2.557860	-76.116670	MT259114
KU 158252	*"Microsciurus" flaviventer*	K	Peru	Loreto	Teniente Lopez, 1.5 km N of	-2.557860	-76.116670	
MVZ 154931	*"Microsciurus" flaviventer*	K	Peru	Amazonas	Vicinity of Huampami (Aguaruna village), Rio Cenepa	-4.455630	-78.161230	MT259118
MVZ 154932	*"Microsciurus" flaviventer*	K	Peru	Amazonas	Vicinity of Huampami (Aguaruna village), Rio Cenepa	-4.455630	-78.161230	MT259119
**AMNH 68149**	*"Microsciurus" flaviventer*	K	Ecuador	Oriente	Rio Suno, abajo g marro	-0.474505	-77.640629	
**AMNH 71608**	*"Microsciurus" flaviventer*	K	Peru	Loreto	Rio Curaray	-2.366667	-74.083333	
LMUSP (ICA 237)	*"Microsciurus" flaviventer*	K	Brazil	Amazonas	Santo Antônio do Içá, margem direita do Rio Içá, oposto à Comunidade São Pedro	-3.038182	-68.879747	MT259113
LSUMZ-M 1565	*"Microsciurus" flaviventer*	K	Peru	Loreto	Quebrada Orán, ca. 5 km N Río Amazonas, 85 km NE Iquitos	-3.199000	-72.706000	
LSUMZ-M 1568	*"Microsciurus" flaviventer*	K	Peru	Loreto	Quebrada Orán, ca. 5 km N Río Amazonas, 85 km NE Iquitos	-3.199000	-72.706000	MT259115
**USNM 267562**	*"Microsciurus" flaviventer*	K	Ecuador	Pastaza	Sara-Yacu	-1.730000	-77.480000	
**AMNH 60464**[a]	*"Microsciurus" sabanillae*	K	Ecuador	Zamora-Chinchipe	Zamora, Sabanilla	-4.033333	-79.016667	
**AMNH 69238**	*"Microsciurus" sabanillae*	K	Peru	Loreto	Requena, Santa Rosa, upper Ucayali River	-4.966667	-73.833333	
USNM 581937	*"Microsciurus" sabanillae*	K	Peru	Amazonas	Cordillera del Condor, Valle Rio Comaina, camp at head of Valley below Table Mountain	-3.877000	-78.413000	MT259123
**USNM 275218**	*Echinosciurus aureogaster*	G	Guatemala	Quiche	Nebaj	15.406794	-91.147297	
**USNM 509015**	*Echinosciurus aureogaster*	G	Mexico	Nayarit	Estanzuela	21.235906	-104.383378	
**USNM 89297**	*Echinosciurus colliaei*	G	Mexico	Nayarit	San Blas	21.538690	-105.271038	
**USNM 96230**	*Echinosciurus colliaei*	G	Mexico	Sonora	Camoa, Rio Mayo	27.228668	-109.256309	MT240882
**USNM 234276**	*Echinosciurus colliaei*	G	Mexico	Distrito Federal	Desierto De Los Leones, 12 mi SE of City of Mexico	19.310727	-99.293345	
**USNM 249863**	*Echinosciurus deppei*	G	Guatemala	Peten	L. Del Sotz	16.982046	-89.692258	
**USNM 337569**	*Echinosciurus deppei*	G	Nicaragua	Matagalpa	Hla Tepeyac	13.017151	-85.834318	
MSB 248241	*Echinosciurus variegatoides*	G	Panama	Colón	Santa Rosa, Aguas Claras	9.166667	-79.666667	MT240884
USNM 449883	*Echinosciurus variegatoides*	G	Panama	Bocas Del Toro	Isla San Cristobal, Bocatorito	9.242064	-82.261056	
USNM 449884	*Echinosciurus variegatoides*	G	Panama	Bocas Del Toro	Isla San Cristobal, Bocatorito	9.242064	-82.261056	MT264767

USNM 449887	*Echinosciurus variegatoides*	G	Panama	Bocas Del Toro	Tierra Oscura, 3.5 Km S. Tiger Key	9.196700	-82.275600	
**USNM 512075**	*Echinosciurus yucatanensis*	G	Mexico	Yucatan	Gramal	20.609951	-89.091315	MT240885
USNM 569025	*Glaucomys volans*	Outgroup	United States	Texas	Harrison County, Longhorn Army Ammunition Plant	32.665714	-94.143014	
USNM 569823	*Glaucomys volans*	Outgroup	United States	Pennsylvania	Berks County, Brecknoch Township, Gouglesville, Hirneisen Farm	40.250000	-76.020000	MT259089
**AMNH 135435**	*Guerlinguetus aestuans* "a"	J	Venezuela	Bolívar	Gran Sabana, Camarata Valley	5.500000	-61.500000	
USNM 599925	*Guerlinguetus aestuans* "a"	J	Guyana		Mount Roraima, N slope	5.283300	-60.750000	MT259077
MPEG 45441	*Guerlinguetus aestuans* "b"	J	Brazil	Pará	Santarém, Comunidade Alto Mentai	-2.800250	-55.583570	MT259078
MPEG (DICO 001)	*Guerlinguetus aestuans* "b"	J	Brazil	Mato Grosso	Reserva Extrativista Guariba-Roosevelt, margem direita Rio Roosevelt	-9.000556	-60.354167	
MPEG (RETA 15)	*Guerlinguetus aestuans* "b"	J	Brazil	Pará	Santarém, Comunidade de Capixauã	-2.612361	-55.192083	MT259079
**AMNH 16949**	*Guerlinguetus aestuans* "c"	J	Venezuela	Bolívar	Cedeno, Suapure	7.233333	-65.166667	
**AMNH 36489**	*Guerlinguetus aestuans* "c"	J	Brazil	Pernambuco	São Lourenço da Mata	-8.000000	-35.050000	MT259088
IEPA 3704	*Guerlinguetus aestuans* "c"	J	Brazil	Amapá	Porto Grande, margem direita do Rio Vila Nova, Floresta Estadual do Amapá	0.470000	-52.010000	MT259084
IEPA 4384	*Guerlinguetus aestuans* "c"	J	Brazil	Pará	Almeirim, margem direita do Rio Jari, Igarapé Pacanari	0.681910	-52.593170	
ISEM-T 1758	*Guerlinguetus aestuans* "c"	J	French Guiana	Petit Saut	Sinnamary, Petit Saut	5.050000	-53.050000	
ISEM-T 5176	*Guerlinguetus aestuans* "c"	J	French Guiana	Cacao	Roura, Cacao	4.576944	-52.468611	MT259083
ISEM-T 6101	*Guerlinguetus aestuans* "c"	J	French Guiana	Cayenne	Camp du Tigre, Cayenne	4.908333	-52.308333	
LMUSP (BM 10678)	*Guerlinguetus aestuans* "c"	J	Brazil	Pará	Vitória do Xingu, margem esquerda do Rio Xingu	-2.873760	-52.015519	
LMUSP (BM 17058)	*Guerlinguetus aestuans* "c"	J	Brazil	Pará	Vitória do Xingu, margem esquerda do Rio Xingu	-2.873760	-52.015519	
LMUSP (ENM 13)	*Guerlinguetus aestuans* "c"	J	Brazil	Amazonas	São Gabriel da Cachoeira, 5º PEF Maturaca	0.634605	-66.124988	MT259085
MN 56819	*Guerlinguetus aestuans* "c"	J	Brazil	Amazonas	Barcelos, Rio Katana-u	1.208611	-64.789167	MT259081
MPEG (CN 029)	*Guerlinguetus aestuans* "c"	J	Brazil	Pará	Faro, Flota de Faro, margem esquerda do Rio Nhamundá	-1.714011	-57.213300	
MPEG (CN 048)	*Guerlinguetus aestuans* "c"	J	Brazil	Pará	Faro, Flota de Faro, margem esquerda do Rio Nhamundá	-1.714011	-57.213300	
MPEG (CN 158)	*Guerlinguetus aestuans* "c"	J	Brazil	Pará	Alenquer, ESEC Grão-Pará, porção sul	-0.165489	-55.186400	
MPEG (CN 160)	*Guerlinguetus aestuans* "c"	J	Brazil	Pará	Alenquer, ESEC Grão-Pará, porção sul	-0.165489	-55.186400	MT259087
MPEG (CN 175)	*Guerlinguetus aestuans* "c"	J	Brazil	Pará	Oriximiná, ESEC Grão-Pará, porção norte	-1.285419	-58.695900	
MPEG (CN 201)	*Guerlinguetus aestuans* "c"	J	Brazil	Pará	Oriximiná, ESEC Grão-Pará, porção norte	-1.285419	-58.695900	MT259086

MPEG (CN 241)	*Guerlinguetus aestuans* "c"	J	Brazil	Pará	Almerim, Reserva Biológica Maicuru	-0.828619	-53.931200	
MPEG (CN 269)	*Guerlinguetus aestuans* "c"	J	Brazil	Pará	Almerim, Flota Paru, margem direita do rio Paru de Leste	-0.943969	-53.236300	
MPEG (CN 270)	*Guerlinguetus aestuans* "c"	J	Brazil	Pará	Almerim, Flota Paru, margem direita do rio Paru de Leste	-0.943969	-53.236300	
MPEG (CN 294)	*Guerlinguetus aestuans* "c"	J	Brazil	Pará	Óbidos, ESEC Grão-Pará, porção central	-0.630281	-55.728500	
MPEG (PECC 03)	*Guerlinguetus aestuans* "c"	J	Brazil	Pará	Marajó-Afuá, Rio Cuieiras, PE Charapucú	-0.226960	-50.590000	
MPEG (PECC 26)	*Guerlinguetus aestuans* "c"	J	Brazil	Pará	Marajó-Afuá, Igarapé Torrão, rio Preto, PE Charapucú	-0.418840	-50.494876	MT259080
UFES-CTA 4256	*Guerlinguetus aestuans* "c"	J	Brazil	Pará	Vitória do Xingu, margem esquerda do Rio Xingu	-3.259346	-51.856208	
UFPA (JMIJ 35)	*Guerlinguetus aestuans* "c"	J	Brazil	Pará	Tapajós	-5.613410	-57.122430	MT259082
CMARF 1568	*Guerlinguetus brasiliensis*	J	Brazil	Bahia	Belmonte, Fazenda Ouro Verde	-15.895070	-39.238440	
CMARF 1569	*Guerlinguetus brasiliensis*	J	Brazil	Bahia	Belmonte, Fazenda Ouro Verde	-15.895070	-39.238440	
FMNH 141601	*Guerlinguetus brasiliensis*	J	Brazil	Sao Paulo	Ilha do Cardoso	-25.133333	-47.966667	MT174525
LMUSP 301	*Guerlinguetus brasiliensis*	J	Brazil	São Paulo	Sorocaba, APP Toyota	-23.374444	-47.470833	
LMUSP (DTM 277)	*Guerlinguetus brasiliensis*	J	Brazil	São Paulo	Caraguatatuba, Parque Estadual da Serra do Mar	-23.581412	-45.484681	MT174527
LMUSP (DTM 41)	*Guerlinguetus brasiliensis*	J	Brazil	São Paulo	Caraguatatuba, Parque Estadual da Serra do Mar	-23.581412	-45.484681	
LMUSP (EFA 41)	*Guerlinguetus brasiliensis*	J	Brazil	Pará	Pacajá, LT Xingu-Estreito	-3.929722	-51.069722	MT174526
**MCN-M 866**	*Guerlinguetus brasiliensis*	J	Brazil	Minas Gerais	Braúna, UHE Porto Estrela	-19.116389	-42.657778	
MCN-M 1388	*Guerlinguetus brasiliensis*	J	Brazil	Pará	Parauapebas, FLONA de Carajás	-6.220806	-50.298406	MT174522
MCN-M 2944	*Guerlinguetus brasiliensis*	J	Brazil	Minas Gerais	Brumadinho, Mina do Córrego do Feijão	-20.113931	-44.115419	
MCNU 3683	*Guerlinguetus brasiliensis*	J	Brazil	Santa Catarina	Anita Garibaldi, margem direita UHE Barra Grande	-27.788560	-51.154506	MT174521
MPEG (ANRA 10)	*Guerlinguetus brasiliensis*	J	Brazil	Pará	Portel, Igarapé Açaituba	-2.224211	-50.544875	MT174528
MPEG (ANRA 12)	*Guerlinguetus brasiliensis*	J	Brazil	Pará	Portel, Igarapé Quirino	-2.166682	-50.644508	
MTR-CIT 1128	*Guerlinguetus brasiliensis*	J	Brazil	Rio Grande do Sul	Itá	-27.256812	-52.392234	MT174518
MTR-CIT 1785	*Guerlinguetus brasiliensis*	J	Brazil	São Paulo	Biritiba Mirim	-23.596572	-46.026164	MT174517
MTR-CIT 1821	*Guerlinguetus brasiliensis*	J	Brazil	São Paulo	Juquitiba	-23.928496	-47.066410	MT174516
MTR-ITM 192	*Guerlinguetus brasiliensis*	J	Brazil	São Paulo	Piedade	-23.716279	-47.422659	MT174520
MTR-ITM 444	*Guerlinguetus brasiliensis*	J	Brazil	São Paulo	Juquitiba	-23.928496	-47.066410	MT174519
MVZ 182070	*Guerlinguetus brasiliensis*	J	Brazil	São Paulo	Ubatuba, Fazenda Capricórnio, 5 km N Ubatuba	-23.416667	-45.116667	MT174515

MVZ 182071	*Guerlinguetus brasiliensis*	J	Brazil	São Paulo	Ubatuba, Praia do Félix	-23.383333	-44.466667	
MVZ 192702	*Guerlinguetus brasiliensis*	J	Brazil	São Paulo	Capão Bonito, Base do Carmo, Fazenda Intervales	-24.333333	-48.416667	
MVZ 200389	*Guerlinguetus brasiliensis*	J	Brazil	São Paulo	São Sebastião, Fazenda da Toca, 2.4 km E, 0.8 km NE (by road) Ilhabela, Ilha de São Sebastião	-23.816667	-45.350000	
MZUSP (GTG 54)	*Guerlinguetus brasiliensis*	J	Brazil	Pará	Santana do Araguaia, Fazenda Fartura	-9.732500	-50.325278	MT174524
MZUSP (GTG 55)	*Guerlinguetus brasiliensis*	J	Brazil	Pará	Santana do Araguaia, Fazenda Fartura	-9.732500	-50.325278	MT174523
UFES-CTA 14	*Guerlinguetus brasiliensis*	J	Brazil	Espírito Santo	Vitória, Parque Estadual da Fonte Grande	-20.350000	-40.350000	
UFES-CTA 85	*Guerlinguetus brasiliensis*	J	Brazil	Bahia	Nova Viçosa, Fazenda Suécia	-17.878889	-40.026389	MT174514
UFES-CTA 119	*Guerlinguetus brasiliensis*	J	Brazil	Espírito Santo	Pancas, Córrego São Bento, Fazenda do Dr. Rolly Luís	-19.225833	-40.761667	MT174513
UFES-CTA 223	*Guerlinguetus brasiliensis*	J	Brazil	Espírito Santo	Águia Branca, Fazenda Pedra Redonda	-18.973333	-40.770556	
UFES-CTA 614	*Guerlinguetus brasiliensis*	J	Brazil	Espírito Santo	Viana, Pimenta	-20.379167	-40.468333	
UFES-CTA 1063	*Guerlinguetus brasiliensis*	J	Brazil	Minas Gerais	Itanhandu, Posses, 13 km SE Itanhandu	-22.383333	-44.850000	MT174512
UFES-CTA 3776	*Guerlinguetus brasiliensis*	J	Brazil	Minas Gerais	Belo Horizonte, Parque das Mangabeiras	-19.945590	-43.910533	MT174511
UFES-CTA 3817	*Guerlinguetus brasiliensis*	J	Brazil	Espírito Santo	Conceição da Barra, Floresta Nacional do Rio Preto	-18.355278	-39.844167	MT174510
**UFSC 274**	*Guerlinguetus brasiliensis*	J	Brazil	Santa Catarina	Leoberto Leal, A. Wagner	-27.621143	-49.292333	
**UFSC 2930**	*Guerlinguetus brasiliensis*	J	Brazil	Santa Catarina	Ipuaçu, AHE Quebra Queixo	-26.657551	-52.547408	
**UFSC 4047**	*Guerlinguetus brasiliensis*	J	Brazil	Santa Catarina	Xaxim, Arvoredo, PCH Arvoredo	-27.040977	-52.466292	MT174509
USNM 549526	*Guerlinguetus brasiliensis*	J	Brazil	Pará	Rio Xingu, east bank	-3.650000	-52.916667	
**AMNH 73917**	*Hadrosciurus* “species 3”	L	Peru	Loreto	Maynas, Orosa, Amazon River	-3.433333	-72.133333	
**AMNH 73924**	*Hadrosciurus* “species 3”	L	Peru	Loreto	Maynas, Orosa, Amazon River	-3.433333	-72.133333	
MUSM 13353	*Hadrosciurus* “species 3”	L	Peru	Loreto	Rio Galvez, Nuevo San Juan	-5.250000	-73.166670	
CBF (LHE 1311)	*Hadrosciurus ignitus*	L	Bolivia	Chuquisaca	El Limón, left bank of Rio Santa Marta	-20.700192	-64.300089	
CBF (LHE 1312)	*Hadrosciurus ignitus*	L	Bolivia	Chuquisaca	El Limón, left bank of Rio Santa Marta	-20.700192	-64.300089	
KU 144563	*Hadrosciurus ignitus*	L	Peru	Madre de Dios	Reserva Cuzco Amazonico, 14 km E of Puerto Maldonado	-12.600000	-69.054490	MT259124
LMUSP (MJ 302)	*Hadrosciurus ignitus*	L	Brazil	Rondônia	Porto Velho, margem esquerda do Rio Madeira, Caiçara	-9.437526	-64.849635	
LSUMZ-M 4467	*Hadrosciurus ignitus*	L	Bolivia	La Paz	Prov. B. Saavedra, 83 km by road E Charazani, Cerro Asunta Pata	-15.083000	-68.550000	MT259125
LSUMZ-M 4471	*Hadrosciurus ignitus*	L	Bolivia	La Paz	Prov. B. Saavedra, 83 km by road E Charazani, Cerro Asunta Pata	-15.083000	-68.550000	
MSB 211524	*Hadrosciurus ignitus*	L	Bolivia	Beni	Totaisal, 1 km SW of Estacion Biologica Del Beni	-14.510000	-66.210000	MT259126

MSB 235873	*Hadrosciurus ignitus*	L	Bolivia	La Paz	Serronia Bella Vista	-15.683300	-67.500000	
MSB 239309	*Hadrosciurus ignitus*	L	Bolivia	La Paz	13.7 km by rd NE from La Reserva	-15.733330	-67.516670	MT259127
MVZ 190350	*Hadrosciurus ignitus*	L	Brazil	Acre	Igarapé Porongaba, right bank Rio Juruá	-8.666667	-72.783333	MT259128
MVZ 190351	*Hadrosciurus ignitus*	L	Brazil	Acre	Nova Vida, right bank Rio Juruá	-8.366667	-72.816667	
MVZ 191206	*Hadrosciurus ignitus*	L	Brazil	Acre	Cruzeiro do Sul, left bank Rio Juruá	-7.633333	-72.600000	
OMNH 29986/4031	*Hadrosciurus ignitus*	L	Argentina	Salta	Iruya, ca. 10 km de la intersección de la Ruta Prov. No. 18 el camino a Cortaderas	-22.990278	-64.690611	
**USNM 255114**	*Hadrosciurus ignitus*	L	Peru	Junín	La Merced	-11.050000	-75.316667	
**USNM 528438**	*Hadrosciurus ignitus*	L	Peru	Madre de Dios	Boca Rio Manu	-12.266667	-70.850000	
**AMNH 33690**	*Hadrosciurus igniventris*	L	Colombia	Caquetá	Morelia, La Murelia	1.516667	-75.683333	
**AMNH 64047**	*Hadrosciurus igniventris*	L	Peru	Cajamarca	Cutervo, Chaupe	-5.166667	-79.166667	
**AMNH 78621**	*Hadrosciurus igniventris*	L	Brazil	Amazonas	São Gabriel do Cachoeira, Tauá, Uaupés River	0.616667	-69.100000	
IDSM (AUATI 78)	*Hadrosciurus igniventris*	L	Brazil	Amazonas	RESEX Auatí-Paraná	-1.934167	-66.232500	
IDSM (AUATI 95)	*Hadrosciurus igniventris*	L	Brazil	Amazonas	RESEX Auatí-Paraná	-1.934167	-66.232500	MT259090
**MPEG 1927**	*Hadrosciurus igniventris*	L	Brazil	Roraima	Rio Mucajaí, sul de Boa Vista	2.420000	-60.870000	MT259091
**USNM 251907**	*Hadrosciurus igniventris*	L	Colombia		Guaicaramo	4.716667	-73.033333	
USNM 599924	*Hadrosciurus igniventris*	L	Venezuela	Amazonas	Neblina Base Camp, Rio Mawarinuma	0.830000	-66.170000	
**AMNH 68271**	*Hadrosciurus pyrrhinus*	L	Ecuador	Napo	Loreto, San José Nuevo	-0.433333	-77.333333	
**AMNH 231771**	*Hadrosciurus pyrrhinus*	L	Peru	Junín	Tarma, 2 mi NW San Ramon	-11.416667	-75.700000	
AMNH 272859	*Hadrosciurus pyrrhinus*	L	Peru	Loreto	Rio Galvez, Nuevo San Juan	-5.250000	-73.166670	
MPEG 41755	*Hadrosciurus pyrrhinus*	L	Brazil	Amazonas	Jutai, RDS Cujubim, P.1., transecto mata de terra firme	-4.654170	-68.323940	MT259092
MPEG 41784	*Hadrosciurus pyrrhinus*	L	Brazil	Amazonas	Jutai, RDS Cujubim, P.2., descendo margem esquerda rio Mutum	-4.935490	-68.173360	MT259093
MPEG (RDSC 101)	*Hadrosciurus pyrrhinus*	L	Brazil	Amazonas	Jutai, RDS Cujubim, baixo rio Mutum, afluente dir. médio rio Jutai, afluente direito alto rio Solimoes, comunidade Pirarucu	-4.670524	-68.132023	MT259096
MUSM 13354	*Hadrosciurus pyrrhinus*	L	Peru	Loreto	Rio Galvez, Nuevo San Juan	-5.250000	-73.166670	MT259094
MVZ 193723	*Hadrosciurus pyrrhinus*	L	Brazil	Amazonas	Colocação Vira-Volta, left bank Rio Juruá on Igarapé Arabidi, affluent of Paranã Breu	-3.283333	-66.233333	MT259095
TTU 98890	*Hadrosciurus pyrrhinus*	L	Peru	Loreto	Maynas, Iquitos, 25 km S, Estacion Biologica Allpahuayo	-3.966670	-73.416670	
TTU 101104	*Hadrosciurus pyrrhinus*	L	Peru	Loreto	Maynas, Iquitos, 25 km S, Estacion Biologica Allpahuayo	-3.966670	-73.416670	

**USNM 255115**	*Hadrosciurus pyrrhinus*	L	Peru	Junín	La Merced	-11.050000	-75.316667	
AMNH 272825	*Hadrosciurus spadiceus*	L	Peru	Loreto	Rio Galvez, Nuevo San Juan	-5.250000	-73.166670	MT259097
IDSM (AUATI 97)	*Hadrosciurus spadiceus*	L	Brazil	Amazonas	RDS Mamirauá	-2.000278	-66.201111	
KU 144566	*Hadrosciurus spadiceus*	L	Peru	Madre de Dios	Reserva Cuzco Amazonico, 14 km E of Puerto Maldonado	-12.600000	-69.054490	MT259101
KU 144567	*Hadrosciurus spadiceus*	L	Peru	Madre de Dios	Reserva Cuzco Amazonico, 14 km E of Puerto Maldonado	-12.600000	-69.054490	
KU 144572	*Hadrosciurus spadiceus*	L	Peru	Madre de Dios	Reserva Cuzco Amazonico, 14 km E of Puerto Maldonado	-12.600000	-69.054490	
LMUSP (EFA 16)	*Hadrosciurus spadiceus*	L	Brazil	Amazonas	Anori, margem esquerda do Rio Purus, Comunidade do Caua-Cuiuanã, proximidades da Comunidade	-4.240372	-61.724023	
LMUSP (EFA 27)	*Hadrosciurus spadiceus*	L	Brazil	Amazonas	Anori, margem esquerda do Rio Purus, Comunidade do Caua-Cuiuanã, Igarapé do Cuiuanã, trilha do Acarizinho	-4.168714	-61.726627	MT259098
LMUSP (EFA 31)	*Hadrosciurus spadiceus*	L	Brazil	Amazonas	Anori, margem esquerda do rio Purus, Comunidade do Caua-Cuiuanã, mata atrás da Comunidade	-4.234045	-61.734642	
LMUSP (ICA 129)	*Hadrosciurus spadiceus*	L	Brazil	Amazonas	Santo Antônio do Içá, margem esquerda do Rio Içá, Comunidade Cuiauá ou Monte Tabor	-2.885465	-68.368681	MT259099
LMUSP (JAP 165)	*Hadrosciurus spadiceus*	L	Brazil	Amazonas	Japurá, margem direita do Rio Japurá, antiga Vila de Santa Fé, trilha da Canoa Virada	-1.764217	-66.357172	MT259100
MPEG 41747	*Hadrosciurus spadiceus*	L	Brazil	Amazonas	Jutai, RDS Cujubim, P.4, a 500m no transecto	-5.638010	-69.187770	MT259103
MPEG 41754	*Hadrosciurus spadiceus*	L	Brazil	Amazonas	Jutai, RDS Cujubim, P.1. Boca do Igarapé Sto. Antonio	-4.654170	-68.323940	MT259104
MPEG 41775	*Hadrosciurus spadiceus*	L	Brazil	Amazonas	Jutai, RDS Cujubim, P.2. Igapó, rio Mutum	-4.935490	-68.173360	MT259105
MPEG 41776	*Hadrosciurus spadiceus*	L	Brazil	Amazonas	Jutai, RDS Cujubim, P.2. Igapó, rio Mutum	-4.935490	-68.173360	MT259106
MPEG 44284	*Hadrosciurus spadiceus*	L	Brazil	Pará	Santarém, Comunidade de Boim	-3.093440	-55.522360	MT259107
MSB 55208	*Hadrosciurus spadiceus*	L	Bolivia	Santa Cruz	San Miguel Rincon	-17.416667	-63.566667	MT259108
MSB 99062	*Hadrosciurus spadiceus*	L	Bolivia	Beni	Totaisal	-14.881111	-66.328056	
MSB 210549	*Hadrosciurus spadiceus*	L	Bolivia	Santa Cruz	San Miguel Rincon	-17.383333	-63.533333	
MVZ 153500	*Hadrosciurus spadiceus*	L	Peru	Amazonas	Chichijam Entsa [=Chichijam Creek], headwaters Rio Huampami, 3 hrs by trail N Huampami	-4.441670	-78.115020	
MVZ 153501	*Hadrosciurus spadiceus*	L	Peru	Amazonas	Kagka (Aguaruna village), Rio Kagka, tributary of Rio Comaina	-4.458980	-78.195050	
MVZ 190352	*Hadrosciurus spadiceus*	L	Brazil	Amazonas	Colocação Sabiá, left bank Rio Juruá	-6.783330	-70.816670	MT259109
MVZ 190354	*Hadrosciurus spadiceus*	L	Brazil	Amazonas	Colocação Sabiá, left bank Rio Juruá	-6.783330	-70.816670	

MVZ 192704	*Hadrosciurus spadiceus*	L	Peru	Madre de Dios	Albergue Cuzco Amazonico	-12.550000	-69.050000	MT259110
MVZ 193724	*Hadrosciurus spadiceus*	L	Brazil	Acre	Ocidente, right bank Rio Juruá	-8.566667	-72.800000	
MVZ 193726	*Hadrosciurus spadiceus*	L	Brazil	Acre	Ocidente, right bank Rio Juruá	-8.566667	-72.800000	MT259111
UFMT (LAB 90)	*Hadrosciurus spadiceus*	L	Brazil	Mato Grosso	Cuiabá, Bairro Coophema	-15.634458	-56.060244	MT259102
USNM 599923	*Hadrosciurus spadiceus*	L	Bolivia	Santa Cruz	El Refugio	-14.767222	-61.034722	
MSB 61161	*Hesperosciurus aberti*	D	United States	Arizona	Graham County, Grahm Mtns. Hospital Flats	32.665100	-109.911000	MT211954
MSB 40646	*Hesperosciurus griseus*	D	United States	California	Mariposa County, 8 mi N, 3 mi E Oakhurst	37.446031	-119.598744	MT211955
USNM 584420	*Hylopetes phayrei*	Outgroup	Myanmar	Mandalay	Pyin-Oo-Lwin (Maymyo), 6.8 mi. ENE, Yangon Monastary	22.067500	96.563500	
**USNM 338172**	*Leptosciurus boquetensis*	H	Panama	Darien	Cerro Mali	8.170000	-77.230000	
**USNM 179564**	*Leptosciurus isthmius*	H	Panama	Darien	Cana	7.730000	-77.680000	
**USNM 292133**	*Leptosciurus isthmius*	H	Colombia	Choco	Baudo Mountains, Rio Jurubida	5.970000	-77.280000	
**USNM 554228**	*Leptosciurus isthmius*	H	Colombia	Valle del Cauca	Beuhaventure, 6 Km N	3.870000	-77.080000	
**USNM 113309**	*Leptosciurus mimulus*	H	Ecuador	Esmeraldas	San Javier	1.070000	-78.780000	
**USNM 113311**	*Leptosciurus mimulus*	H	Ecuador	Esmeraldas	Carondelet	1.100000	-78.700000	MT259072
**USNM 309028**	*Leptosciurus mimulus*	H	Colombia	Narino	La Guayacana	1.430000	-78.450000	
**USNM 499514**	*Leptosciurus otinus*	H	Colombia	Antioquia	Zaragoza, 23 Km S, 22 Km W, at Providencia	7.500000	-74.866667	
**USNM 499515**	*Leptosciurus otinus*	H	Colombia	Antioquia	Zaragoza, 25 Km S, 22 Km W, at La Tirana	7.500000	-74.866667	MT259073
**USNM 271319**	*Leptosciurus pucheranii*	H	Colombia	Santander	Virolín, Santander Sur, 28 Km S of Charala, Diutama Road	6.083333	-73.200000	
**USNM 293776**	*Leptosciurus pucheranii*	H	Colombia	Antioquia	La Bodega, S Side Rio Negrito, Highway Sonson-Narino	5.700000	-75.116667	MT240886
**AMNH 32497**	*Leptosciurus similis*	H	Colombia	Cauca	El Tambo, La Gallera	2.583333	-76.916667	MT259074
**USNM 303846**	*Leptosciurus similis*	H	Colombia	Cauca	Cerro Munchique	2.530000	-76.980000	
**USNM 292132**	*Microsciurus* "species 1"	G	Colombia	Choco	Rio Nuqui, Baudo Mountains, Base	5.670000	-77.270000	
**USNM 318364**	*Microsciurus alfari*	G	Panama	Panama	Candelaria Hydrographic Station, Rio Pequeni	9.370000	-79.530000	
**USNM 338164**	*Microsciurus alfari*	G	Panama	Darien	Cerro Tacarcuna	8.170000	-77.300000	
USNM 575652	*Microsciurus alfari*	G	Panama	Bocas Del Toro	Nuri	8.913025	-81.815461	
USNM 575653	*Microsciurus alfari*	G	Panama	Bocas Del Toro	Nuri	9.395119	-82.531526	
USNM 570626	*Neosciurus carolinensis*	F	United States	Wisconsin	Portage County, Stevens Point, 2633 Ellis Street, 54481	44.521300	-89.560900	MT240881

**USNM 329635**	*Parasciurus alleni*	E	Mexico	Nuevo Leon	El Potosi	24.874760	-100.237306	
MSB 157846	*Parasciurus arizonensis*	E	United States	New Mexico	Grant County, Gila River near Spar Canyon	33.023350	-108.535650	MT240880
MSB 47449	*Parasciurus nayaritensis*	E	United States	Arizona	Cochise County, Chiricahua Mts, 2 mi SW Paradise	31.914200	-109.242400	MT240883
USNM 568615	*Parasciurus niger*	E	United States	Maryland	Montgomery County, Potomac River, south side of Watkins Island	29.042200	-77.279200	
USNM 570471	*Parasciurus niger*	E	United States	Maryland	Dorchester County, Vienna, ca. 1.5 mi SSW on Elliots Island Road	38.469200	-75.845300	
**USNM 55607**	*Parasciurus oculatus*	E	Mexico	Hidalgo	Tulancingo	20.121236	-98.359043	
**USNM 55933**	*Parasciurus oculatus*	E	Mexico	Mexico	Volcano Toluca, N Slope	19.127644	-99.754869	
**USNM 197262**	*Rheithrosciurus macrotis*	B	Indonesia	Borneo	Kalimantan, Sungai Menganne	0.490000	117.570000	
**USNM 488087**	*Rheithrosciurus macrotis*	B	Malaysia	Sabah	Mount Kinabalu National Park, Ranau District, Poring	6.000000	116.680000	
**USNM 152749**	*Sciurus anomalus*	C	Turkey		Soumela	40.686465	39.655032	
**USNM 140867**	*Sciurus lis*	C	Japan	Saitama Prefecture	Musasi, Titibu	35.990278	139.076389	MT134013
MSB 148785	*Sciurus vulgaris*	C	Russia	Sakha Republic	Kenkeme River, 40 km W Yakutsk	62.070030	128.938310	MT211956
LSUMZ-M 936	*Simosciurus nebouxii*	I	Peru	Piura	Pariñas, 7 km N, 15 km E Talara	-4.533000	-81.150000	
MVZ 196054	*Simosciurus nebouxii*	I	Peru	Cajamarca	2.5 km N (by air) Monte Seco, Rio Zana	-7.110517	-79.516486	MT259075
**AMNH 34686**	*Simosciurus stramineus*	I	Ecuador	Guayas	Daule	-1.833333	-79.933333	
**AMNH 62886**	*Simosciurus stramineus*	I	Ecuador	Los Ríos	Vinces	-1.533333	-79.750000	MT259076
**AMNH 66640**	*Simosciurus stramineus*	I	Ecuador	Guayas	Santa Elena, Cerro Manglar Alto	-1.833333	-80.733333	
**AMNH 131723**[b]	*Syntheosciurus brochus*	G	Costa Rica	Alajuela	Poás, Volcan Poás	10.198092	-84.198861	MT240890
**AMNH 71613**	*Syntheosciurus granatensis*	G	Ecuador	Pichincha	Quito, La Carolina	-0.216667	-78.500000	MT240887
**MUSM 23157**	*Syntheosciurus granatensis*	G	Peru	Cajamarca	1 km S Hito Jesus	-4.895000	-78.895278	
TTU 102463	*Syntheosciurus granatensis*	G	Ecuador	El Oro	Near to La Victoria, road from Arenillas to Puyango	-3.550000	-80.066670	
**USNM 48689**	*Syntheosciurus granatensis*	G	Nicaragua		Escondido River, 2.5 mi from Bluefields	12.051127	-83.760050	
**USNM 113314**	*Syntheosciurus granatensis*	G	Ecuador	Esmeraldas	Pambilar	0.766667	-79.083333	
**USNM 334696**	*Syntheosciurus granatensis*	G	Colombia	Valle del Cauca	Rio Raposo, Colombia Pacific Coast Virology Field Station	3.716667	-77.133333	
**USNM 442017**	*Syntheosciurus granatensis*	G	Venezuela	Zulia	Nr. Cerro Azul, 33 Km NW La Paz	10.850000	-72.250000	
**USNM 461886**	*Syntheosciurus granatensis*	G	Trinidad and Tobago	Tobago	Runnemede	11.250000	-60.700000	MT240888
USNM 449881	*Syntheosciurus granatensis*	G	Panama	Bocas Del Toro	Tierra Oscura, 3.5 Km S. Tiger Key	9.196700	-82.275600	

USNM 464871	*Syntheosciurus granatensis*	G	Panama	Bocas Del Toro	Isla Colón, La Gruta	9.400000	-82.266667	
USNM 464872	*Syntheosciurus granatensis*	G	Panama	Bocas Del Toro	Isla Colón, La Gruta	9.400000	-82.266667	
USNM 575630	*Syntheosciurus granatensis*	G	Panama	Bocas Del Toro	Nuri	8.913025	-81.815461	
USNM 575637	*Syntheosciurus granatensis*	G	Panama	Bocas Del Toro	Nuri	8.913025	-81.815461	MT240889
USNM 578377	*Syntheosciurus granatensis*	G	Panama	Bocas Del Toro	Peninsula Valiente, Punta Alegre	9.162550	-81.905133	
USNM 578378	*Syntheosciurus granatensis*	G	Panama	Bocas Del Toro	Peninsula Valiente, Punta Alegre	9.162550	-81.905133	
MSB 273400	*Tamiasciurus douglasii*	A	Mexico	Baja California	Sierra San Pedro Martir, 15 mi E Meling Ranch	30.905278	115.501111	
USNM 570572	*Tamiasciurus hudsonicus*	A	United States	Wisconsin	Portage County	44.333400	-89.381400	
USNM 570573	*Tamiasciurus hudsonicus*	A	United States	Wisconsin	Portage County	44.333400	-89.381400	

[a]Holotype of *Microsciurus sabanillae* Anthony, 1920.

[b]Holotype of *Syntheosciurus poasensis* (Goodwin, 1942), considered as junior-synonym of *S. brochus* by Thorington et al. (2012).
