# Supplementary material for: Museomics of tree squirrels: a dense taxon sampling of mitogenomes reveals hidden diversity, phenotypic convergence, and the need of a taxonomic overhaul
Source: BMC Evol Biol. 2020 Jun 26;20:77. doi: 10.1186/s12862-020-01639-y (PMC7320592; doi:10.1186/s12862-020-01639-y)
Supplement: Supplementary file 7 — Additional file 7. State coding used on ancestral state reconstruction analyses. Number of upper premolars were coded as one (1) or two (2), and number of pairs of mammae were coded as three (3), four (4), or five (5). Taxonomic identifications follow the new arrangement proposed here (see text for detailed explanation). [file 12862_2020_1639_MOESM7_ESM.pdf]

Additional file 7

State coding used on ancestral state reconstruction analyses. Number of upper premolars were coded as one (1) or two (2), and number of pairs of mammae were coded as three (3), four (4), or five (5). Taxonomic identifications follow the new arrangement proposed here (see text for detailed explanation).

Species	Upper premolars	Pairs of mammae
"*Microsciurus*" "species 2"	2	3
"Microsciurus" flaviventer	2	3
"Microsciurus" sabanillae	2	3
Echinosciurus aureogaster	2	4
Echinosciurus colliaei	2	4
Echinosciurus deppei	2	3
Echinosciurus variegatoides	2	4
Echinosciurus yucatanensis	2	4
Guerlinguetus aestuans "a"	1	4
Guerlinguetus aestuans "b"	1	4
Guerlinguetus aestuans "c"	1	4
Guerlinguetus brasiliensis	1	4
Hadrosciurus "species 3"	1	4
Hadrosciurus ignitus	1	3
Hadrosciurus igniventris	1	4
Hadrosciurus pyrrhinus	1	4
Hadrosciurus spadiceus	1	4
Hesperosciurus aberti	2	4
Hesperosciurus griseus	2	4
Leptosciurus boquetensis	2	3
Leptosciurus isthmius	2	3
Leptosciurus mimulus	2	3
Leptosciurus otinus	2	3
Leptosciurus pucheranii	1	3
Leptosciurus similis	2	3
Microsciurus "species 1"	2	3
Microsciurus alfari	2	3
Neosciurus carolinensis	2	4

Parasciurus alleni	1	4
Parasciurus arizonensis	1	4
Parasciurus nayaritensis	1	4
Parasciurus niger	1	4
Parasciurus oculatus	1	4
Rheithrosciurus macrotis	1	3
Sciurus anomalus	1	5
Sciurus lis	2	3
Sciurus vulgaris	2	4
Simosciurus nebouxii	1	4
Simosciurus stramineus	1	4
Syntheosciurus brochus	2	3
Syntheosciurus granatensis	1	3
Tamiasciurus douglasii	2	4
Tamiasciurus hudsonicus	2	4
